# Impact of *Bacillus* spp. spores and gentamicin on the gastrointestinal microbiota of suckling and newly weaned piglets

**DOI:** 10.1371/journal.pone.0207382

**Published:** 2018-11-27

**Authors:** Ann-Sofie Riis Poulsen, Nadieh de Jonge, Jeppe Lund Nielsen, Ole Højberg, Charlotte Lauridsen, Simon M. Cutting, Nuria Canibe

**Affiliations:** 1 Immunology and Microbiology, Department of Animal Science, Aarhus University, Tjele, Denmark; 2 Center for Microbial Communities, Department of Chemistry and Bioscience, Aalborg University, Aalborg, Denmark; 3 School of Biological Sciences, Royal Holloway, University of London, Egham, Surrey, United Kingdom; University of Illinois, UNITED STATES

## Abstract

Administrating antibiotics to young piglets may have short- and long-term consequences on the gut microbiota. We hypothesised that these consequences may be alleviated by concurrent probiotic administration. The study objective was to investigate the effect of administrating gentamicin and a mixture of *Bacillus (B*.*) licheniformis*, *B*. *subtilis* and *B*. *amyloliquefaeceans* spores on the gut microbiota of piglets pre- and post-weaning. Twenty-four sows and their litters were randomly allocated to four treatment groups receiving; a) *Bacillus* spore mixture (six *B*. *subtilis*, two *B*. *amyloliquefaeceans*, and one *B*. *licheniformis*) fed to sows and piglets (PRO); b) gentamicin (5 mg per day) administered to piglets on day 4, 5, and 6 of age (AB); c) *Bacillus* spore mixture fed to sows and piglets, and gentamicin to piglets (PRO+AB); or d) no administration of probiotics or antibiotics (CTRL). Faecal and digesta samples were collected repeatedly during the study. Selected samples were subjected to 16S rRNA gene sequencing, culture counts, and organic acid, biogenic amine and tissue gene expression analysis. Treatment had a significant effect on the faecal microbial community composition on day 28 and 42, and colonic community on day 28. Faecal species richness (observed and estimated) and Shannon index, and colonic species richness, were higher in AB compared to PRO piglets on day 28, and were not significantly different from day 42. PRO piglets had the highest faecal concentration of iso-butyric acid on day 7 and a higher butyric acid concentration compared to CTRL piglets. We conclude that gentamicin and *Bacillus* spores influence the gut microbial diversity of piglets, although administration of gentamicin did not result in dysbiosis as hypothesised.

## Introduction

The newborn young has an immature immune system and is hence highly susceptible to infections. Newborn piglets are in high risk of developing diarrhoea in their first week of life and the condition often leads to antibiotic treatment, even though there is no clear evidence of the potential infectious agent(s) involved [1]. However, administering antibiotics to newborn piglets may have short- and long-term consequences on the developing gastrointestinal microbiota and the local immune system, which may ultimately result in increased disease susceptibility later in life.

Microbial gut colonisation begins during birth when the newborn comes into contact with microbes from the mother and the surrounding environment. This initial colonisation leads to an array of complex processes responsible for establishing the gut microbiota [2]. The gut microbiota plays a pivotal role in maturing the gut and local immunological functions [3]. During early life, however, the gut microbiota is instable and highly susceptible to disturbances by influencing factors, antibiotics being one of them [4]. Administering antibiotics within the first week of life has been shown to cause short- and long-term changes in the composition of the gut microbiota and on intestinal gene expression related to immunological functions [5, 6]. Moreover, human studies have reported an association between antibiotic treatments in early life and immune-related disorders [7]. Alleviating the negative effect of antibiotics on the immature gut microbiota might be crucial in improving both intestinal and general health of piglets.

Probiotics are defined as ‘live microbial feed supplements which beneficially affect the host animal by improving its intestinal microbial balance’ [8] and are believed to exert their anti-pathogenic effects by competing with pathogens for intestinal mucosal binding sites and nutrients, and inhibiting pathogen growth by producing organic acids and antibiotic-like compounds [9]. Bacteria commonly used as porcine probiotics belong to the *Lactobacillus*, *Enterococcus*, and *Bacillus* genera. Unlike most other probiotics, *Bacillus* spp. is a spore-forming bacterium, and hence more resistant to unfavorable environmental conditions than probiotics found as vegetative cells only [10]. *Bacillus cereus* var. Toyoi has been reported to reduce the intestinal number of enterotoxigenic *Escherichia coli*, diarrhoea incidence, and morbidity in weaned piglets [11–13]. Furthermore, it has been reported to have immunological effects by increasing the number of T-lymphocytes locally in the intestinal tissue and faecal IgA concentration [13, 14]. Also *B*. *licheniformis* and *B*. *subtilis* have been found to decrease morbidity, mortality, and post-weaning diarrhoea [15]. Human studies, focusing on the use of probiotics in relation to antibiotic treatment, have reported *Lactobacillus casei* to reduce the incidence of antibiotic-associated diarrhoea and to counteract the effect of antibiotics on the diversity of the gut microbiota [16].

However, detailed information on the effects of early life antibiotic therapy and concomitant probiotic administration on the gut microbiota and local immune parameters in piglets is lacking. The aim of the current study was therefore to investigate the effect of administrating antibiotics and probiotics on the gut microbiota in piglets. We hypothesise that administration of gentamicin, a broad-spectrum aminoglycoside, changes the microbial community of the gut, and that the expected negative effects of antibiotic administration are alleviated by concomitant administration of *Bacillus* spp. spores.

## Materials and methods

### Study design

The present study was conducted according to a license obtained from the Danish Animal Experiments Inspectorate, Ministry of Food, Agriculture and Fisheries, Danish Veterinary and Food administration, license number 2013−15−2934−00822. Sows and piglets were housed according to the general guidelines on housing of pigs before and after farrowing, and after weaning.

A total of 288 piglets (crossbred [Danish Landrace x Yorkshire] x Duroc; mixed females and males) from 24 sows of varying parities were included in the study. All sows originated from Christiansminde Multisite K/S, Denmark. The study was conducted in three blocks with eight sows each, resulting in six replicate sows per treatment. Before farrowing, sows were randomly divided into four treatment groups (two sows per treatment in each block): (a) Sows fed a probiotic mixture from ten days pre-partum until 28 days post-partum, and their piglets fed the same probiotic mixture during lactation (0–28 days of age) and post-weaning (28–42 days of age) (PRO); (b) Sows with piglets were administered gentamicin orally at 4, 5, and 6 days of age (AB); (c) Sows fed a probiotic mixture from ten days pre-partum until 28 days post-partum, and their piglets fed a probiotic mixture during lactation (0–28 days of age) and post-weaning (28–42 days of age), and administered gentamicin orally at 4, 5, and 6 days of age (PRO+AB); (d) Neither sows nor their piglets were fed probiotics or antibiotics (CTRL).

No creep-feed was allowed pre-weaning. The piglets were weaned at 28 days of age and housed litter-wise, with no bedding material. The animals were allowed ad libitum access to a standard weaner diet free from zinc oxide. The study ended two weeks post-weaning. Clinical conditions and occurrence of diarrhoea were recorded daily. Diarrhoea was recorded per litter and scored as 0, 1 or 2. The score 0 was given when no diarrhoea was observed in a litter, score 1 when a mild diarrhoea was observed, and score 2 when there was severe diarrhoea.

### Probiotics

The probiotic mixture consisted of a total of nine *Bacillus* strains, six *Bacillus subtilis*, two *Bacillus amyloliquefaeceans*, and one *Bacillus licheniformis*. Strains were isolated from human faeces by heat treatment (68°C for 1 h) of faecal suspensions and plating at 37°C (aerobic culture for 48 h). This approach ensured that only aerobic spore forming bacteria were cultured. Colonies were isolated at random and the species determined by *gyrA* sequence analysis [17]. None of the strains showed antibiotic resistance in performed MIC assays.

The ‘exhaustion method’ was used for spore production [18]. Each of the nine strains was grown for ~24h in DSM sporulation medium using an Electrolab bioreactor to produce spores. Spores were then harvested by centrifugation, heat-treated (68°C for 45 min) to kill residual vegetative cells and purified as described [18]. Spores were stored at room temperature as lyophilized powders until use, where they were suspended in distilled water and further diluted before fed to the animals. All spores were found in equal amounts in the solution containing all nine spore strains. Sows were fed 1x10^10^ cfu/kg feed two times daily from ten days pre-partum until the piglets were weaned. Piglets were orally administered 2x10^9^ cfu/day on day 3, 5, 7, 10, 13, 16, 20, 24, and 28 of age; 4x10^9^ cfu per day at 33 days of age, and 8x10^9^ cfu/day at 38 days of age. Piglets not receiving probiotics were administered an equal volume of water.

### Antibiotics

Piglets were orally administered 5 mg gentamicin (Gentocin Vet.) as a single bolus on day 4, 5, and 6 of age. Piglets not receiving the antibiotic were administered an equal volume of water.

### Sample and data collection

Piglets were weighed weekly. Faecal samples, directly from the rectum, were collected weekly from three piglets in each litter. Two piglets from each litter were euthanised at 3 days of age and one piglet from each litter at 28 and 42 days of age. Three-day-old piglets were euthanised according to the Danish guideline on euthanasia of piglets weighing less than 5 kg, approved by the Danish Veterinary and Food administration. 28 and 42 days old piglets were euthanized using a captive bolt gun followed by bleeding. The abdomen was incised and the gastrointestinal tract removed. Luminal content (digesta) from the stomach, small intestine (proximal and distal segments), caecum and colon (proximal, middle, and distal segment) was collected immediately after killing. Collected digesta from the two 3-day-old piglets was pooled. pH was measured and the digesta was weighed and taken to the laboratory for further analysis. Bacterial enumeration by culture was performed on digesta samples from the stomach, distal small intestine, caecum, and mid colon. Faecal and digesta subsamples were stored at -20°C for organic acid and biogenic amine analyses. Other faecal and digesta subsamples were snap-frozen in liquid nitrogen and stored at -80°C for 16S rRNA gene amplicon sequencing. An intestinal tissue sample from 50% of the length of the distal small intestine was carefully rinsed with PBS, cut into two pieces of 1 cm^2^ and stored in Dulbecco’s modified Eagle’s medium (DMEM) on ice until stimulation with lipopolysaccharide (LPS).

### DNA extraction

Samples for DNA extraction included 214 faecal samples (day 7, 28 and 42; two day-7 samples from the PRO group were missing) from 72 piglets and 144 digesta samples (distal small intestine and mid colon from day 3, 28, and 42) from 24 piglets. DNA was extracted with the E.Z.N.A. stool DNA Kit (Omega Bio-Tek, inc., VWR international) following their standard protocol for extracting DNA from faecal material, with the following exceptions: In step 2, 450 μL SLX-Mlus buffer was added followed by 2x20 s bead beating (FastPrep FP120; Bio 101 Savant/MP Biomedicals, USA). In step 3, 50 μL DS Buffer and Proteinase K solution were added and followed by bead beating for 20 s. Step 4 was followed by centrifugation at 2,000 g for 30 s. In step 5, 170 μL SP2 buffer was added and followed by bead beating for 20 s. If no supernatant was present after step 8, another 180 μL SLX-Mlus, 20 μl DS buffer and 67 μL SP2-buffer were added and the samples vortexed for 30 s., put on ice for 3 min and centrifuged at maximum speed for 5 min. DNA extract purity was evaluated with Nanodrop ND1000 (Thermo Scientific, USA) and quantified fluorometrically with Qubit 3.0 HS dsDNA assay (Life Technologies, Thermo Fisher Scientific, USA). DNA concentrations were normalized to 5 ng/μL.

### 16S rRNA gene amplicon sequencing

Amplicon libraries were generated by targeted amplification of the V1-V3 hypervariable regions of the bacterial 16S rRNA gene. The PCR reaction (25 μl) contained 10 ng template DNA, Platinum High Fidelity buffer (1x), dNTP (400 uM of each), MgSO_4_ (1.5 mM), and Platinum Taq DNA polymerase High Fidelity (1U), and barcoded library adapters (400 nM) (V1-V3 primers: 27F AGAGTTTGATCCTGGCTCAG and 534R ATTACCGCGGCTGCTGG). Thermocycler settings: Initial denaturation at 95°C for 2 min, 30 cycles of 95°C for 20 s, 56°C for 30 s, 72°C for 60 s, and final elongation at 72°C for 5 min. PCR reactions were run in duplicate for each sample and pooled before purification. Purification of the amplicon libraries was performed using the Agencourt AMPure XP bead protocol (Beckman Coulter, USA) and eluted in 23 μL nuclease-free water. Individual libraries were quantified with Quant-iT HS dsDNA assay (Life Technologies, USA) and quality checked on a Tapestation 2200 (Agilent, USA). Libraries were pooled in equimolar concentrations, and diluted to 4 nM. The library pool was sequenced using a MiSeq (Illumina, USA) and MiSeq reagent kit v3 (2x300 PE). Raw reads are available in the Sequence Read Archive [19] under accession number PRJNA503676.

### Amplicon bioinformatic processing and analysis

The obtained raw sequencing reads were quality filtered and trimmed using trimmomatic (v0.32) [20], only keeping reads with a minimum length of 275 bp. The trimmed reads were merged using FLASH v. 1.2.7 [21] and read pairs between 425 and 525 bp in length were formatted for use with the UPARSE workflow [22]. Reads were dereplicated and clustered into Operational Taxonomical Units (OTUs) using USEARCH7 at 97% sequence similarity. Taxonomy was assigned using the RDP-classifier as implemented in QIIME [23] with a minimum confidence of 0.8 and Greengenes (version 08–2013) as a reference database. Results were analysed in R (version 3.2.2 for Mac) using R studio (version 0.99.489 for Mac) and the *Ampvis* package [24].

### Organic acid and biogenic amine analysis

The concentrations of short-chain fatty acids and lactic acid in faeces and digesta samples were measured as previously described by [25]. Biogenic amine concentrations (agmatine, putrescine, cadaverine and tyramine) were measured according to the following procedure. Samples (including a blank sample) were diluted 10-fold with an internal standard solution, 111.11 mg 2-aminoheptanoic acid/L dissolved in 0.1 M HCl, with a final concentration in the sample of 100 mg/L, and homogenized in a Smasher paddle blender (bioMérieux Industry, USA) for 2 min. Proteins were precipitated by mixing 20 μL sample or standard mix containing 60 mg putrescine dihydrochloride/L, 70 mg cadaverine dihydrochlorine/L, 70 mg tyramine hydrochloride/L, 150 mg agmatine sulfate salt /L, and 100 mg 2-aminoheptanoic acid (internal standard)/L, dissolved in 0.1 M HCl, with 780 μL 0.1 M hypochloric acid and 240 μL 2 M perchloric acid, followed by incubation at room temperature for 5 min, and centrifugation at 15.000 x g for 5 min. Derivates were produced by adding 320 μL 0.5 M sodium bicarbonate to 200 μL supernatant from the protein precipitation step, followed by the addition of 600 μL 5 mM Fmoc-Cl solution and mixing. The samples were subsequently heated at 40°C for 10 min, followed by mixing with 40 μL concentrated hypochloric acid. Samples were centrifuged at 15.000 x g for 5 minutes. 600 μL supernatant were transferred to a vial and analysed on a Agilent HPLC 1200 serie equipped with vacuum degasser, binary pump, auto sampler with thermostat, column thermostat, and fluorescence detector. The used LC column was Kinetex Reversed Phase 2.6u C18 100Å, 100x4.6mm, which was heated to 40°C. The fluorescence detector had the excitation and emission wavelengths set at 265 and 315 nm, respectively. Samples were placed in the auto sampler at 5°C and 3.0 μl were injected. Flow was set at 1.0 ml/min in a gradient run with solvent A 80/20 v/v % 20 mM ammonium acetate buffer pH 3.3/acetonitrile and solvent B 90/10 v/v % acetonitrile/ultra pure water. The gradient program was as follows: solvent B was held at 25% from 0 to 2 min, increased to 80% B over 16 min, increased to 86% B over 2 min, a rapid increase to 100% B for 0.1 min, and hold at 100% B for 1.9 min, then a rapid decreased to 25% B for 1 min, and a final hold at 25% for 2 min. Chromatograms were integrated using Agilent ChemStation software.

### Microbiological analysis of digesta samples

For microbial plating, digesta samples (approximately 5 g) were transferred to bottles containing 50 ml pre-reduced salt medium [26]. The bottle content was then transferred to a CO_2_-flushed bag and homogenized in a Smasher paddle blender for 2 min. One ml digesta homogenate was transferred to a Hungate tube containing 9 ml pre-reduced salt medium and 10-fold dilutions were prepared using the technique previously described by [27]. The samples were plated on selective (and indicative) and non-selective agar plates.

*Enterobacteriaceae* were enumerated on MacConkey agar (Merck 1.05465) after aerobic incubation for 1 day. Yeasts were enumerated on Malt, Chlortetracycline and -Amphenicol agar (Merck 1.03753 (yeast extract), 1.05397 (malt extract), 1.07224 (bacto-pepton), 1.08337 (glucose), 1.01614 (agar-agar), and Oxoid Sr0177E) after aerobic incubation for 2 days. Hemolytic bacteria were enumerated on blood agar (Oxoid Pb5039A) after aerobic incubation for 1 day. *Clostridium perfringens* were enumerated using the pour-plate technique on Tryptose Sulfit Cycloserine agar (Merck 1.11972, 1.00888) after anaerobic incubation for 1 day. Lactic acid bacteria were enumerated on de Man, Rogosa and Sharpe agar (Merck 1.10660) after anaerobic incubation for 2 days. Total anaerobic bacteria were enumerated in roll tubes containing pig colon fluid-glucose-cellobiose agar [26] and incubated for 7 days. For *Bacillus* spore enumeration, the samples were incubated in a water bath at 80°C for 10 min. *Bacillus* spores were enumerated on Casein soya bean digest broth agar (Oxoid CM0129) after aerobic incubation for 1 day. All plates and roll-tubes were incubated at 37°C.

### LPS-stimulation of ileal tissue

One 1 cm^2^ tissue sample was transferred to 1 ml DMEM and 10 μl PBS (control) and the second 1 cm^2^ tissue sample was transferred to 1 ml DMEM and 10 μl LPS (1 μg/μl; Sigma L4391). The two test tubes were incubated at 37°C for 120 min under constant gently shaking. After ended incubation, the tubes were placed on ice for 10 min and the tissue then transferred to 700 μl RNAlater (Sigma-Aldrich), stored at 5°C for one day and then stored at -20°C until gene expression analysis.

### Gene expression analysis

The intestinal tissue samples were transferred to a 2 ml Eppendorf tube together with a 5 mm stainless steel bead and homogenized on the TissueLyser system (Qiagen). Total mRNA for gene expression analysis was extracted using the NucleoSpin RNA isolation kit (Macherey-Nagel, Germany) following the manufacturers protocol. The Nanodrop ND1000 was used for measuring RNA quantity and assessing RNA quality (Thermo Scientific, USA). All samples were diluted to 100 ng/μl and converted to cDNA using the High-Capacity cDNA Reverse Transcription kit (Applied Biosystems, USA) according to the manufacturer’s protocol. Real Time PCR reactions contained 2 μl cDNA and 8 μl mix containing primers, probe (Table 1) and 2X TaqMan mastermix, and run in triplicate on the Applied Biosystems ViiA 7 Real-Time PCR system (Life Technologies) with the following thermocycler settings: 50°C for 2 min, 95°C for 10 min, and 40 cycles of 95°C for 15 s and 60°C for 60 s. Glyceraldehyde-3-phosphate dehydrogenase (GAPDH) was used as housekeeping gene. The Applied Biosystem ViiA 7 software was used for determining the gene expression cycle threshold (Ct) values. Relative quantification (comparative threshold method) was used for evaluating the gene expression level using non-LPS stimulated ileal samples from 28 days old CTRL pigs as reference samples. For each sample the Ct value of the GAPDH gene was subtracted from the Ct value of the target gene (ΔCt). The average ΔCt value of the reference animals was then subtracted from the ΔCt value of all samples (ΔΔCt). The expression of the target gene was given as fold change calculated by 2^-ΔΔCt^.

**Table 1:** Information on primers, probes and genes for real-time PCR.

Target gene	Primer sequences	Accession number
GAPDH^1^	Forward: 5’-GTCGGAGTGAACGGATTTGG-3’ Reverse: 5’-CAATGTCCACTTTGCCAGAGTTAA-3’ Probe: 5’-CGCCTGGTCACCAGGGCTGCT-3’	AF017079
TNF-α^2^	Forward: 5’-AACCCTCTGGCCCAAGGA-3’ Reverse: 5’-GGCGACGGGCTTATCTGA-3’ Probe: FAM-TCAGATCATCGTCTCAAAC-MGB	X57321
COX-2^3^	Forward: 5’-GGGACGATGAACGGCTGTT-3’ Reverse: 5’-CACAATCTTAATCGTTTCTCCTATCAGT-3’ Probe: 5’-AGACGAGCAGGCTGA-3’	NM_214321
IL-10^4^	Forward: 5’-GAGGAGGTGAAGAGTGCCTTTA-3’ Reverse: 5’-CTCACCCATGGCTTTGTAGACA-3’ Probe: FAM-CCTCTCTTGGAGCTTGC-MGB	L20001
ZO-1^5^	TaqMan Gene Expression Assay. catalogue no. 4351370. assay ID ss03373514_m1 (Applied Biosystems. Thermo Fisher Scientific)	
OCLN^6^	TaqMan Gene Expression Assay. catalogue no. 4351370. assay ID ss03377507_u1 (Applied Biosystems. Thermo Fisher Scientific)	
CLDN-2^7^	TaqMan Gene Expression Assay. catalogue no. 4351370. assay ID ss03375002_u1 (Applied Biosystems. Thermo Fisher Scientific)	
CLDN-4^8^	TaqMan Gene Expression Assay. catalogue no. 4351370. assay ID ss03375006_u1 (Applied Biosystems. Thermo Fisher Scientific)	

^1^GAPDH = glyderaldehyde-3-phosphate dehydrogenase;

^2^TNF-α = tumour necrosis factor-α;

^3^COX-2 = cyclooxygenase-2;

^4^IL-10 = interleukin-10;

^5^ZO-1 = zonula occludens-1;

^6^OCLN = occludin;

^7^CLDN-2 = claudin-2;

^8^CLDN-4 = claudin-4

### Statistical analysis

Principal Component Analysis was performed on square root transformed OTU abundances. Significance of treatment was tested on the first two principal components (PCs) using the envfit parametric test and on the Bray-Curtis dissimilarity matrix using the Adonis test. A constrained (by treatment) redundancy analysis was included as a visual tool to clarify potential grouping of treatment groups [28].

The impact of treatment and age on bacteria counts, organic acid and biogenic amine concentration, microbial diversity (Shannon index) and species richness (observed and estimated chao1 index), gene expression levels, body weight, and the number of days with diarrhoea were investigated by fitting the data to a linear mixed model using the *lmer* function from the *lme4* package [29] using R (version 3.2.2 for Mac) and R studio (version 0.99.489 for Mac). Diet, age, and gut segment, and the interactions between diet and age, and diet, age and gut segment were included as fixed effects, while piglet and sow were included as random effects (by including random intercept terms) to account for multiple observations from the same litter and the same pig. When analysing the body weight variable, piglet birth body weight was included as a co-variate. The fixed effects were tested using an F-test with Kenward-Roger approximation, where the reduced model was tested against the full model. This was done using the *KRmodcomp* function in the *pbkrtest* package [30]. When a fixed effect was found to be significant, a post-hoc test was performed using the *multcomp* package and Bonferroni adjustment to correct for multiple comparisons [31]. Effects were considered significant when *p*<0.05.

## Results

### Concentration of Bacillus spp. in administered solutions

Multiple solutions containing the nine different *Bacillus* spore strains were prepared during the experiment, and administered to the piglets and sows. From each solution, a subsample was used for verifying the number of spores. The spore solutions used for the piglets contained in average 1.23x10^9^ cfu/ml and the solutions used for the sows contained in average 2.91x10^9^ cfu/ml.

### General observations

The weekly body weight of the piglets is shown in Table 2. Body weight was not affected by treatment (*p =* 0.89) or gender (*p* = 0.72). During the suckling period, the pens with piglets belonging to the PRO treatment were found to have the highest number of days with score 2 (severe) diarrhoea, and were different from the CTRL group (p = 0.03), but not from the pens with piglets belonging to the AB and PRO+AB treatments (Table 3). No significant differences in the number of days with score 1 (mild) and score 2 (severe) diarrhoea were found between the four treatment groups during the post-weaning period.

**Table 2:** Piglet bodyweight.

	Treatment group^2^								*p*-value		
Days of age	CTRL		AB		PRO		PRO+AB		T^3^	A^4^	T*A
0	1.5	(1.4–1.6)	1.5	(1.4–1.6)	1.5	(1.4–1.6)	1.5	(1.4–1.6)	0.89	<0.001	<0.001^5^
7	2.7	(2.6–2.9)	2.8	(2.6–2.9)	2.8	(2.6–3.9)	2.5	(2.4–2.7)			
14	5.0	(4.7–5.4)	4.9	(4.6–5.3)	4.9	(4.5–5.2)	4.7	(4.4–5.0)			
21	7.5	(7.0–8.0)	7.0	(6.5–7.5)	6.9	(6.4–7.4)	7.0	(6.5–7.5)			
28	9.7	(9.0–10.4)	9.2	(8.5–9.8)	8.6	(8.0–9.2)	9.4	(8.8–10.1)			
35	9.9	(9.3–10.7)	9.5	(8.9–10.2)	9.1	(8.5–9.8)	9.6	(9.0–10.3)			
42	11.3	(10.6–12.2)	10.8	(10.0–11.6)	10.3	(9.6–11.0)	11.1	(10.4–11.9)			

Piglet body weight (kg) at birth and 7, 14, 21, 28, 35, and 42 days of age^1^

^1^ Values are presented as least square means and 95% confidence intervals (in parentheses).

^2^ CTRL = control; AB = antibiotic group; PRO = probiotic group; PRO+AB = probiotic+antibiotic group.

Number of observations: CTRL = 414; AB = 395; PRO = 414; PRO+AB = 386.

^3^ T = treatment group

^4^ A = age.

^5^ Multiple comparisons with bonferroni correction show that treatment group has no significant effect on piglet body weight. The effect of age is, as expected, highly significant and a comparison of the different days has therefore been left out.

**Table 3:** Number of days with diarrhoea.

	Treatment group^2^				*p*-value		
Diarrhoea score^3^	CTRL	AB	PRO	PRO+AB	T^4^	P^5^	T*P
Score 1					0.19	0.29	0.33
Suckling	5.2 (3.0–7.4)	3.8 (1.7–6.0)	6.3 (4.1–8.5)	6.6 (4.5–8.8)			
Weaned	6.2 (4.1–8.4)	4.9 (2.7–7.1)	7.4 (5.2–9.5)	7.7 (5.5–9.9)			
Score 2					0.03	0.05	0.02
Suckling	0 ^a^ (0–1.3)	0.4 ^a^^b^ (0–1.6)	2.6 ^b^^A^ (1.4–3.9)	2.0 ^a^^b^ (0.7–3.3)			
Weaned	1.2 (0–2.4)	1.0 (0–2.3)	1.2 ^B^ (0–2.4)	1.7 (0.4–2.9)			

Diarrhoea^1^ (number of days) during the suckling (28 days) and post-weaning (14 days) period registered per pen.

^1^ Values are presented as least square means and 95% confidence intervals (in parentheses)

^2^ CTRL = control; AB = antibiotic group; PRO = probiotic group; PRO+AB = probiotic+antibiotic group.

^3^ Score 1 = mild diarrhoea. Score 2 = Severe diarrhoea.

^4^ T = treatment group.

^5^ P = period; suckling or post-weaning period.

^a,b^ Values with different superscripts within a row are significantly different.

^A,B^: Values with different superscripts within a column and score are significantly different.

### Gut microbiota–culture results

**Fig 1:**
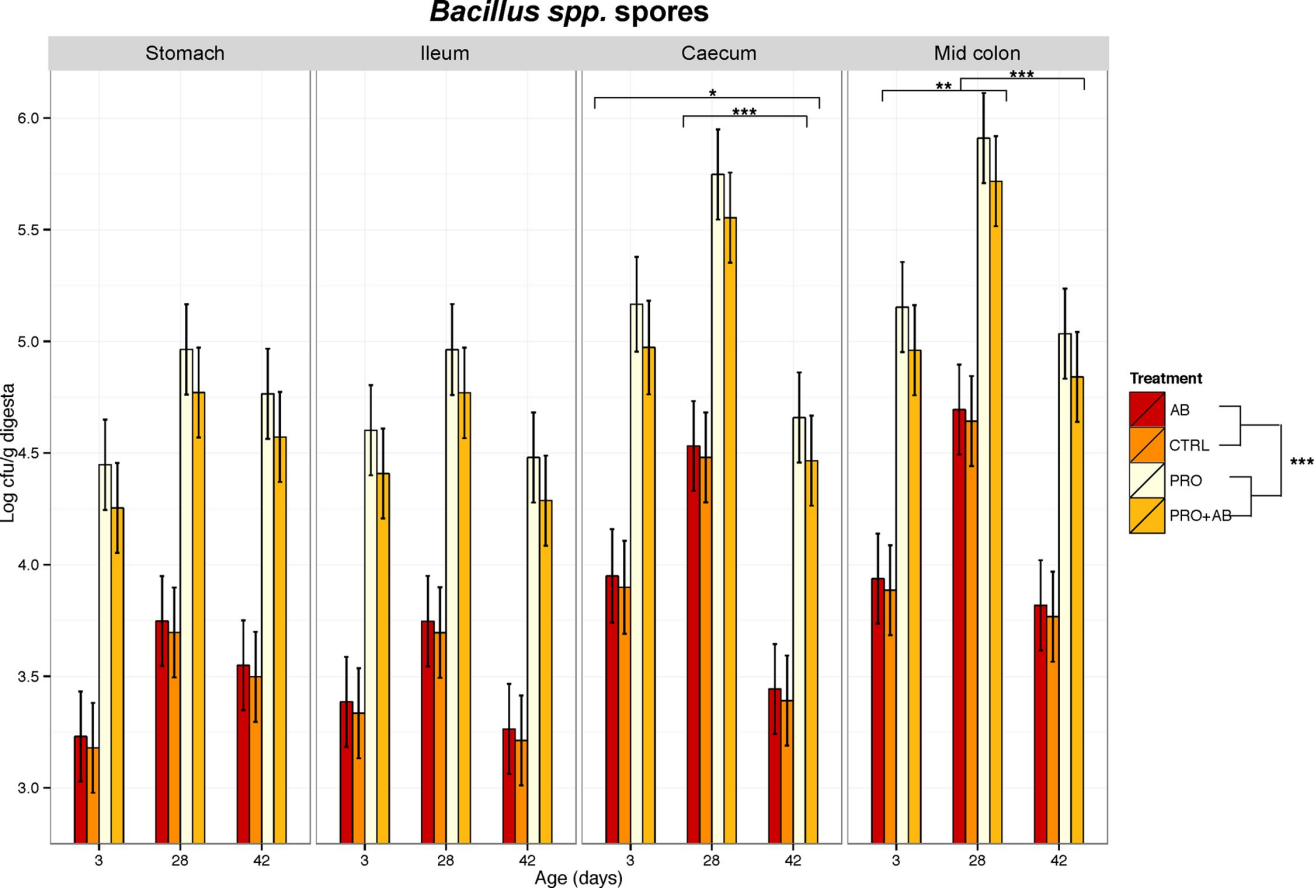
Microbial culture of *Bacillus* spores. *Bacillus* spores in digesta (log cfu/g wet weight) from stomach, ileum, caecum, and mid colon, sampled on day 3, 28, and 42 of age from piglets administered gentamicin (AB; n = 71), piglets administered *Bacillus* spores (PRO; n = 68), piglets administered both gentamicin and *Bacillus* spores (PRO+AB; n = 71), and control piglets receiving neither gentamicin nor *Bacillus* spores (CON; n = 72). Values are presented as least-square means and the 95% confidence intervals presented as vertical bars. Bars embraced by horisontal brackets market by *(0.01≤*p*<0.05), **(0.001≤*p*<0.01) or ***(*p*<0.001) are significantly different.

The treatment factor had a significant effect on the number of *Bacillus* spores along the gastrointestinal tract, with the highest numbers found in PRO and PRO+AB piglets compared to AB and CTRL piglets (*p*<0.001) (Fig 1). In addition, the number of *Bacillus* spp. spores in the caecum was higher on day 3 and 28 than on day 42 (*p*≤0.04), and in the mid colon, it was higher on day 28 (*p*≤0.003) than on day 3 and 42. There were no significant differences among the treatments on the number of *Enterobacteriaceae*, haemolytic bacteria, *C*. *perfringens*, total anaerobic bacteria, or lactic acid bacteria in any of the investigated gut segments (S1 Fig). Treatment had no effect on pH in any of the investigated segments (S1 Table).

### Bacterial metabolites

Age and intestinal segment were found to have an interacting effect on the ileal and mid colonic digesta concentrations of cadaverine and putrescine (S2 Table), and organic acids (S3 Table), while none of the treatmentshad a significant effect. While the biogenic amine concentration was highest in the colon when piglets were 3-days old, it was highest in the ileum when they were 42-days old. Agmatin and tyramin were not included in the analysis as the majority of the observations were below detection level. Ileal and colonic concentrations of cadaverine and putrescine were consistently lower (numerically) in AB piglets compared to the other three groups. Colonic concentrations of propionic acid on day 28 and 42 were unexpectedly higher than the acetic acid concentrations.

Organic acid concentrations in faecal samples from day 7, 28, and 42 are shown in Table 4. PRO piglets had the highest faecal concentration of iso-butyric acid on day 7 (*p* = 0.04) and a higher concentration of butyric acid compared to CTRL piglets also on day 7 (*p* = 0.02). Treatment had no other significant effect on faecal organic acid concentrations.

**Table 4:** Short-chain fatty acids in faeces.

	Treatment group^2^									*p*-values		
Age	CTRL		AB		PRO		PRO+AB		#	T^3^	A^4^	T*A
Acetic acid										0.32	<0.001	0.42
7	28.8	(23.8–33.7)	24.6	(19.7–29.5)	29.0	(24.2–33.9)	29.0	(24.2–33.8)	a			
28	34.9	(30.2–39.6)	30.7	(26.0–35.3)	35.2	(30.4–39.9)	35.1	(30.4–39.7)	b			
42	59.9	(55.3–64.6)	55.7	(51.1–60.4)	60.2	(55.5–64.9)	60.1	(55.4–64.8)	c			
Propionic acid										0.76	<0.001	0.03
7	8.6^A^	(4.7–12.4)	7.8^A^	(4.1–11.5)	11.6^A^	(8.0–15.2)	7.8^A^	(4.3–11.2)				
28	14.2^A^	(10.9–17.5)	10.8^A^	(7.5–14.1)	14.9^A^^B^	(11.4–18.4)	12.9^A^	(9.6–16.2)				
42	24.8^B^	(21.5–28.0)	24.5^B^	(21.2–27.8)	19.5^B^	(16.2–22.8)	25.4^B^	(22.0–28.8)				
Butyric acid										0.84	<0.001	0.004
7	3.7^A^^b^	(0.6–6.9)	5.4^A^^a^^b^	(2.4–8.5)	10.2^a^	(7.3–13.2)	5.2^A^^a^^b^	(2.4–8.1)				
28	8.2^A^^B^	(5.4–10.9)	5.2^A^	(2.4–7.9)	7.6	(4.7–10.4)	6.6^A^	(3.8–9.3)				
42	12.1^B^	(9.4–14.9)	11.2^B^	(8.4–13.9)	8.0	(5.3–10.7)	11.7^B^	(8.9–14.5)				
Valeric acid										0.52	0.11	0.09
7	2.2	(1.6–2.8)	1.9	(1.3–2.5)	2.4	(1.8–3.0)	2.1	(1.5–2.7)				
28	2.7	(2.1–3.3)	2.4	(1.8–2.9)	2.9	(2.3–3.4)	2.6	(2.0–3.1)				
42	2.7	(2.2–3.3)	2.4	(1.9–3.0)	2.9	(2.3–3.5)	2.6	(2.0–3.2)				
Iso-butyric acid										0.63	0.91	0.01
7	1.6^a^	(0.9–2.4)	1.6^a^	(0.9–2.4)	3.1^A^^b^	(2.4–3.8)	1.7^a^	(1.0–2.4)				
28	2.3	(1.6–3.0)	1.8	(1.1–2.4)	2.3^A^^B^	(1.6–3.0)	2.0	(1.3–2.7)				
42	2.1	(1.5–2.8)	2.4	(1.8–3.1)	1.8^B^	(1.1–2.4)	2.2	(1.6–2.9)				
Iso-valeric acid										0.68	0.29	0.03
7	1.3	(0.6–2.0)	1.4	(0.8–2.1)	2.6^A^	(1.9–3.3)	1.4	(0.8–2.0)				
28	2.2	(1.5–2.8)	1.7	(1.0–2.3)	2.1^A^^B^	(1.5–2.8)	1.8	(1.2–2.4)				
42	1.6	(1.0–2.2)	1.9	(1.3–2.5)	1.3^B^	(0.7–1.9)	1.7	(1.1–2.4)				

Short-chain fatty acids^1^ (mmol/ kg sample) in faeces from piglets at 7, 28, and 42 days of age.

^1^ Values are presented as least square means and 95% confidence intervals (in parentheses).

^2^ CTRL = control; AB = antibiotic group; PRO = probiotic group; PRO+AB = probiotic+antibiotic group.

Number of piglets: CTRL = 49; AB = 50; PRO = 49; PRO+AB = 51.

^3^ T = treatment group.

^4^ A = age.

a, b, c: Rows with different letters are significantly different (*p*<0.05).

^a,b^ Values with different superscripts within a row are significantly different.

^A,B^: Values with different superscripts within a column are significantly different.

### 16S rRNA gene amplicons

Sequencing of 356 samples yielded a total of 10,638,599 reads. A sequencing depth of 10,000 reads was considered appropriate from rarefaction curves, which excluded 23 samples from further analysis. Recovered reads were clustered into 3400 OTUs and classified into 20 phyla, 100 families and 191 genera.

### Faeces

**Fig 2:**
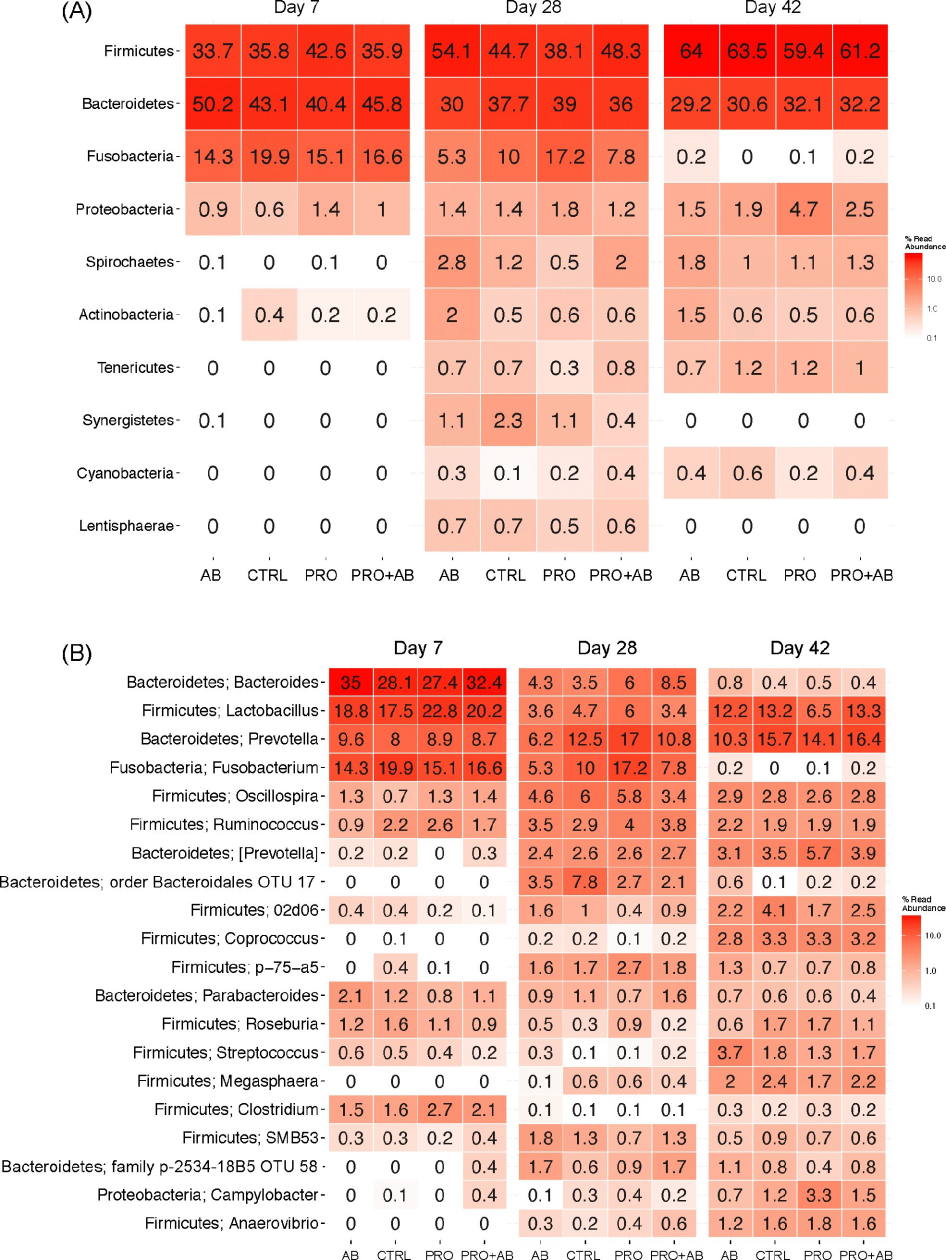
Heatmaps showing bacterial phyla and genera; faecal samples. Heatmaps of faecal samples collected at 7, 28, and 42 days of age from piglets administered gentamicin (AB; n = 53), *Bacillus* spores (PRO; n = 50), both gentamicin and *Bacillus* spores (PRO+AB; n = 53), and control piglets not receiving gentamicin or *Bacillus* spores (CTRL; n = 51). Heatmaps show the relative abundances (%) of (A) the ten most abundant phyla and (B) the 20 most abundant genera in faecal samples. Colours represent the relative abundances on a logarithmic scale.

**Fig 3:**
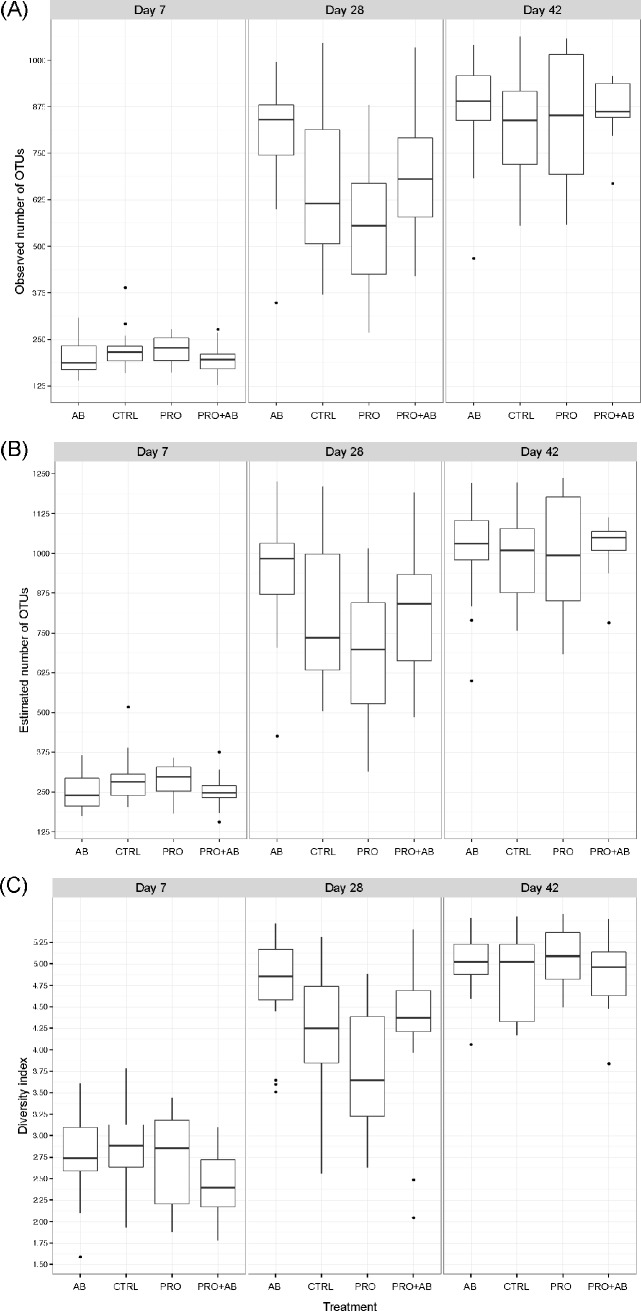
Species richness and diversity; faecal samples. Observed and estimated species richness and Shannon diversity index of faecal samples collected at 7, 28, and 42 days of age from piglets administered gentamicin (AB; n = 53), *Bacillus* spores (PRO; n = 50), both gentamicin and *Bacillus* spores (PRO+AB; n = 53), and control piglets receiving neither gentamicin nor *Bacillus* spores (CTRL; n = 51). Boxplots show the (A) observed and (B) estimated species richness and (C) Shannon diversity index.

Fig 2 shows the relative abundance of the ten most abundant phyla and 20 most abundant genera in faecal samples collected on day 7, 28, and 42 of age. *Firmicutes* and *Bacteroidetes*, followed by *Fusobacteria*, were the most abundant phyla on day 7 and 28 irrespective of treatment. *Firmicutes*, followed by *Bacteroidetes*, were the most abundant phyla on day 42, whereas *Fusobacteria* was detected at very low levels (Fig 2A). At genus level, *Bacteroides*, *Lactobacillus*, *Prevotella and Fusobacterium* were the most abundant genera on day 7; *Prevotella and Fusobacterium* the most abundant on day 28; and *Lactobacillus and Prevotella* the most abundant on day 42 irrespective of treatment. Twelve of the most abundant genera belonged to *Firmicutes*, six to *Bacteroidetes*, one to *Fusobacteria*, and one to *Proteobacteria*. The observed microbial community richness (Fig 3A) and Shannon index (Fig 3C) were lower in PRO piglets compared to AB piglets (*p* = 0.0001; *p*<0.001) and PRO+AB piglets (*p* = 0.03; *p* = 0.046) on day 28; while the estimated richness (Fig 3B) was lower in PRO piglets compared to AB piglets (*p* = 0.0004).

There was a significant effect of treatment on the overall community composition of the four treatment groups on day 28 (*p*_adonis_ = 0.003) and 42 (*p*_adonis_ = 0.008) (S2B and S2D Fig). The results of a redundancy analysis on data from day 28 and 42 are shown in S2C and S2E Fig. Age (*p*_adonis_ = 0.001) (S3 Fig) and litter (*p*_adonis_ = 0.002) had a significant effect on the faecal microbial composition.

### Digesta

**Fig 4:**
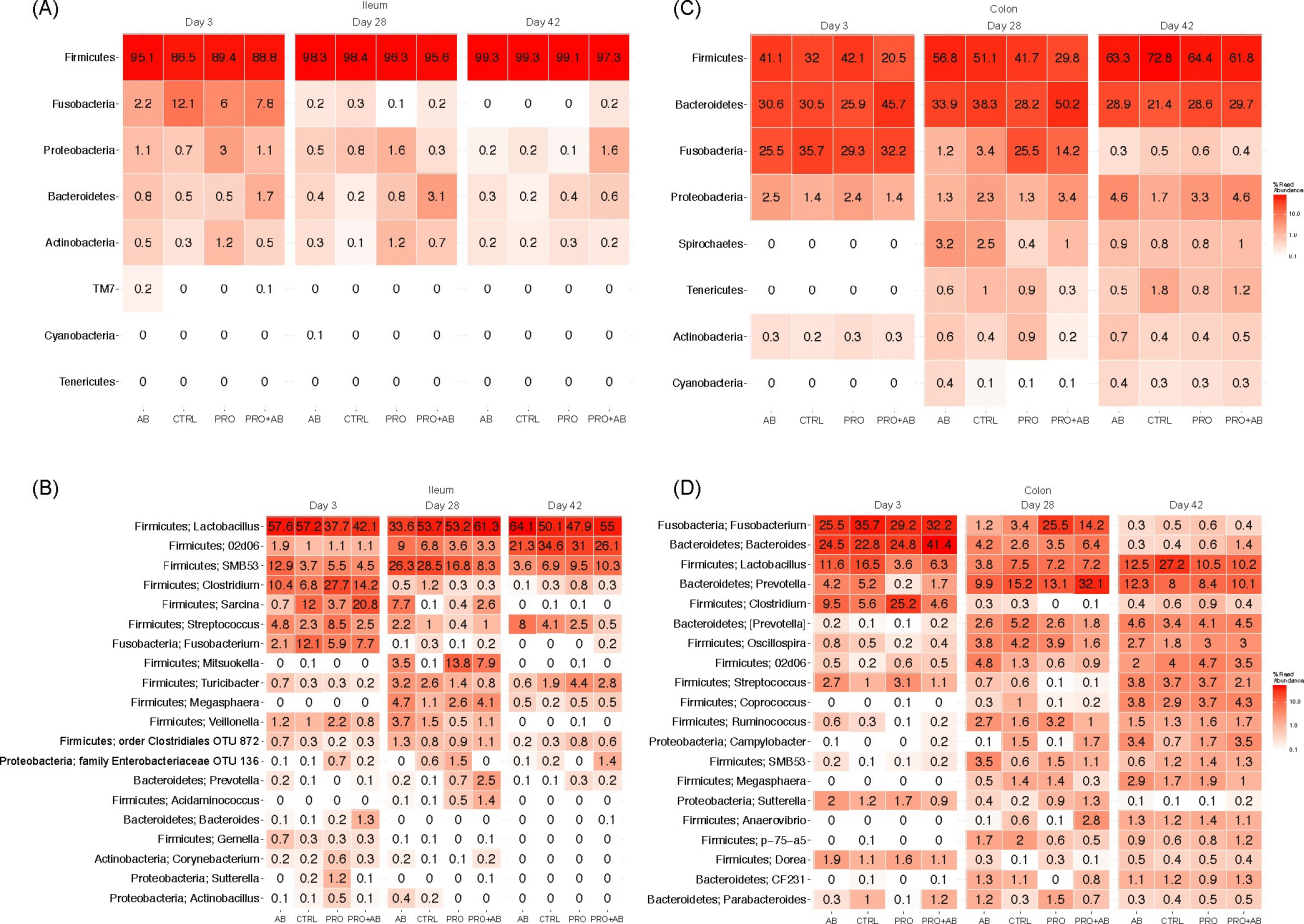
Heatmaps showing bacterial phyla and genera; digesta samples. Heatmaps of ileal and colonic digesta samples collected at 3, 28, and 42 days of age from piglets administered gentamicin (AB; n = 33), *Bacillus* spores (PRO; n = 33), both gentamicin and *Bacillus* spores (PRO+AB; n = 31), and control piglets receiving neither gentamicin nor *Bacillus* spores (CTRL; n = 29). Heatmaps show the relative abundances (%) of (A) the eight most abundant phyla in ileal digesta, (B) the 20 most abundant genera in ileal digesta, (C) the eight most abundant phyla in colonic digesta, and (D) the 20 most abundant genera in colonic digesta. Colours represent the relative abundances on a logarithmic scale.

Fig 4A and 4B show the relative abundances of the eight most prevalent phyla and 20 most prevalent genera in ileal digesta from day 3, 28, and 42 of age. *Firmicutes* was clearly the most abundant phylum at all three days investigated, irrespective of treatment. On day 3, *Fusobacteria* was measured to be between 2.2 and 12.1% in all treatments. *Lactobacillus* was the most abundant genus irrespective of age and treatment. On day 3, SMB53 (family *Clostridiaceae*), *Clostridium*, *Sarcina*, *Streptococcus*, and *Fusobacterium* were also abundant, with the relative abundance of *Sarcina* ranging between 0.7% (AB group) and 20.8% (PRO+AB group). On day 28, *SMB53*, *02d06* (family *Clostriciaceae)*, *Sarcina*, *and Mitsuokella* were the most abundant after *Lactobacillus*, with the relative abundance of *Mitsoukella* ranging between 0.1% (CTRL group) and 13.8% (PRO group). On day 42, 02d06, SMB53, and *Streptococcus* were the most abundant after *Lactobacillus*.

**Fig 5:**
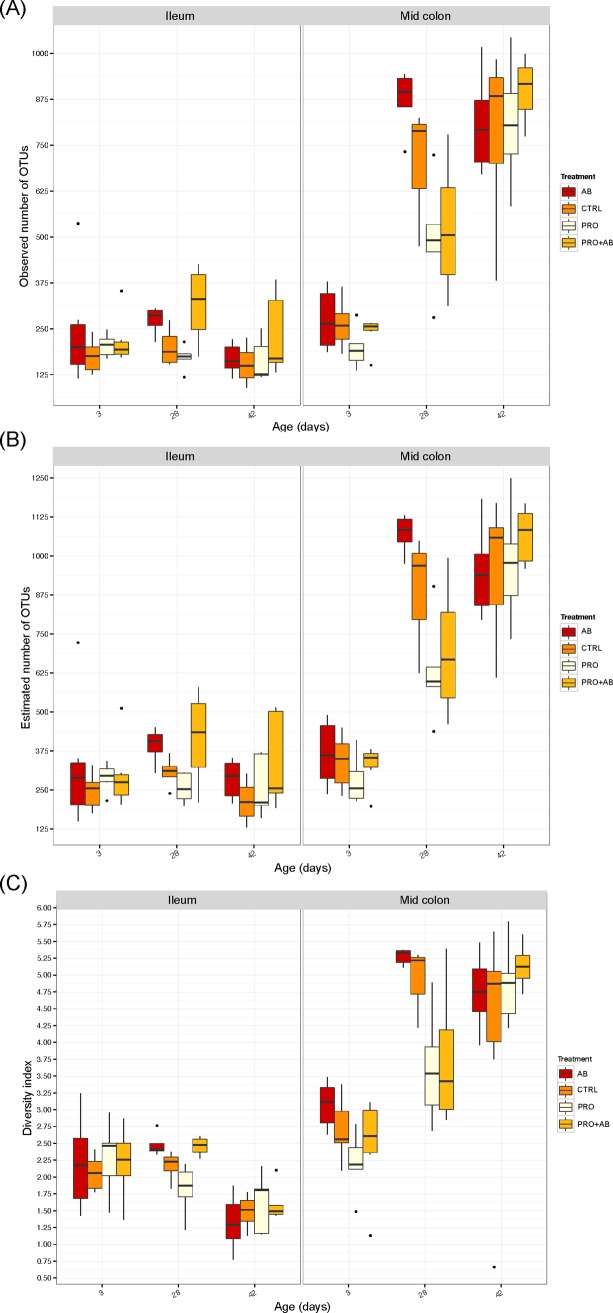
Species richness and diversity; digesta samples. Observed and estimated species richness and Shannon diversity index of ileal and colonic digesta samples collected at 3, 28, and 42 days of age from piglets administered gentamicin (AB; n = 33), *Bacillus* spores (PRO; n = 33), both gentamicin and *Bacillus* spores (PRO+AB; n = 31), and control piglets receiving neither gentamicin nor *Bacillus* spores (CON; n = 29). Boxplots show the (A) observed and (B) estimated species richness, and (C) Shannon diversity index.

Fig 4C and 4D show the relative abundance of the eight most prevalent phyla and 20 most prevalent genera in digesta from mid colon on day 3, 28, and 42 of age. *Firmicutes*, *Bacteroidetes*, and *Fusobacteria* were the most abundant phyla on day 3 irrespective of treatment, while *Firmicutes* and *Bacteroidetes* were the most abundant phyla on day 28 and 42 irrespective of treatment. *Fusobacteria* was also abundant on day 28 in PRO and PRO+AB piglets. At genus level, *Fusobacterium*, *Bacteroides*, *Lactobacillus*, and *Clostridium* were the most abundant genera on day 3 in all treatment groups. *Prevotella*, *Lactobacillus*, and *Fusobacterium* dominated on day 28, with *Fusobacterium* being most abundant in PRO and PRO+AB piglets, and *Prevotella* most abundant in PRO+AB piglets. *Lactobacillus* and *Prevotella* were the dominating genera on day 42, with the highest abundance of *Lactobacillus* in CTRL piglets. The observed richness of colonic digesta on day 28 was higher in AB piglets compared to PRO (*p* = 0.001) and PRO+AB (*p* = 0.02) piglets (Fig 5A), and the estimated richness higher in AB compared to PRO (*p =* 0.001) piglets (Fig 5B).

None of the treatments had an effect on the overall microbial community composition in the mid colon on day 28 (*p*_adonis_ = 0.013) (S4 Fig). Otherwise, treatment had no significant effect on the microbial community. Age had an effect on the overall microbial community in the ileum and mid colon (*p* = 0.001) (S5 Fig). Samples from the ileum and mid colon clustered closer together on day 3 compared to day 28 and 42. Litter had no effect on the microbial community of the ileum and mid colon.

### Ileal gene-expression

Comparative expression of TNF-α, COX-2, IL-10, ZO-1, OCLN, CLDN-2, and CLDN-4 genes are shown in S6 Fig. LPS stimulation of ileal tissue increased the expression of TNF-α (*p* = 0.05) and COX-2 (*p* = 0.04), but had no effect on IL-10, ZO-1, OCLN, CLDN-2, and CLDN-4 gene expression. Treatment had no effect on the gene expression level of any of the investigated genes. TNF-α (*p*<0.001), CLD-4 (*p* = 0.05), and ZO-1 (*p* = 0.01) genes were found to be expressed at higher levels on day 28 compared to day 42.

## Discussion

The potential of using probiotics has been intensively investigated in both humans and animals. In pigs, several probiotics have been tested and some have been found to have beneficial effects on the gut microbial community and intestinal immunological parameters [11, 13, 32]. However, knowledge on the effect of probiotics after therapeutic administration of antibiotics, which can disrupt the gastrointestinal ecology, on the gut microbiota and intestinal health of pigs is lacking.

Analysis of 16S rRNA gene sequences confirmed an effect of treatment on the overall colonic and faecal microbial communities at the day of weaning (day 28), where AB piglets, compared to PRO piglets, consistently had the most diverse microbiota. Contrary to the other treatment groups, the microbial diversity of AB piglets was the same on day 28 as on day 42 (lower on day 28 for the other treatment groups). It could be speculated that these observations indicate an accelerated microbial gut colonisation in the distal gut segments of AB piglets caused by gentamicin inhibiting bacterial groups that would colonise the gut under normal (non-treated) circumstances, thereby allowing a more diverse community to colonise faster. In accordance with these results, [33] suggested an accelerated maturation of the faecal microbiota in tylosin-supplemented pigs. [6] suggests that the effects of intrinsic factors, e.g. the early impact of an antibiotic, are most apparent at times when the gut microbiota has stabilized, as seen just prior to weaning and several weeks post-weaning, and our results are generally supportive of this, as we observed the most significant treatment effect on day 28 and 42, but not on day 7. Interestingly, though, the ileal microbiota was the most stable community across treatments without any significant effects of treatment on the overall community or microbial diversity at 28 and 42 days of age. Well aware of the varying mode of action among antibiotics, subcutaneous tulathromycin injection at four days of age has been shown to have short- and long-term effects on the jenunal microbiota of piglets by decreasing the microbial diversity [5, 6]. The short-term effect was seen when the piglets were 8 days of age, the long-term effect was seen at 176 days of age, while no effect was seen at 55 days of age. So, if the ileal digesta had been collected a few days after antibiotic administration in the current study, we would perhaps have found gentamicin to have a more profound effect on the ileal microbiota. Another explanation might be that the spectrum of activity of gentamicin merely did not cover the community found in the ileum.

Enumerating specific bacterial groups by culture did not show any differences between treatment groups. As gentamicin is especially active against gram-negative bacteria [34], we did expect a reduction in the number of *Enterobacteriaceae* cultured from intestinal digesta. A possible explanation is that the digesta samples were collected 22 and 36 days after the last gentamicin dose was administered, thus providing enough time for the piglets to be re-colonised. Surprisingly, daily administration of subtherapeutic doses of virginiamycin has been shown to decrease counts of total anaerobic bacteria and lactobacilli, and increase counts of coliforms [35].

In the current study, we found the effect of gentamicin to be unexpectedly low. Oral antibiotics are generally thought to cause intestinal dysbiosis, as seen in e.g. humans where diarrhoea is a common complication to antibiotic therapy [16, 36]. However, different classes of antibiotics target different spectra of bacteria, and the development of antibiotic-associated diarrhoea has been shown to be dependent on the type of antibiotic used [37]. In line with this, the present results indicate that the magnitude of the expected dysbiosis caused by antibiotic treatment of pigs, and thereby, the immediate and possible consequences of this later in life, will vary depending on the antibiotic used.

*Bacillus* spores were included to assess whether they were able to counteract the effect of early gentamicin administration, that is, whether the spores could re-establish the microbiota after having been disrupted. However, the relatively low effect of gentamicin both on parameters describing the microbiota composition and activity, and intestinal cellular barrier function, may have impeded the detection of potential effect(s) of *Bacillus* spores on the gut microbial community in the current study. On the other hand, some results do indicate some kind of effect of the antibiotic and counteraction by the bacilli. The concentration of biogenic amines in ileum and colon, although not significantly different among treatment groups, was highest in the PRO group and lowest in the group receiving the antibiotic (AB), increasing again by adding the bacilli (PRO+AB). These data only showed numerical tendencies, and therefore should be interpreted with caution. To allow piglets to be exposed to the spores already from birth, they were included in the sow feed from ten days before expected farrowing. Plate cultures of *Bacillus* spores showed that piglets in the PRO and PRO+AB groups had the highest concentration of spores along the length of their gastrointestinal tracts already on day 3 post-farrowing compared to piglets in the CTRL and AB groups. Probiotic administration started on day 3 after faecal collection, therefore the results show that the spores had been vertically transferred from the sow to the piglets, which is in accordance with [12]. Our results suggest that probiotics administered to the dam during late gestation are transferred to the offspring through contact with maternal faeces.

Piglets given the *Bacillus* spores (PRO and PRO+AB groups) had higher spore counts in their intestinal digesta than the other groups. The applied method quantified only the number of spores and not vegetative cells, and we were not able to account for the precise degree of spore germination. Nevertheless, earlier studies have reported that *B*. *subtilis* and *B*. *licheniformis* are able to germinate in the gut of grower pigs, though only showing limited ability to grow [10]. Due to the resilient spore structure [38], 16S rRNA gene sequencing was expected to detect germinated cells only. The detected abundances of *Bacillus* genera in the ileum, colon, and faeces were, however, extremely low regardless of sample type, age and treatment group. If only a small percentage of the spores germinated, the detection threshold of the sequencing method may have been too high compared to more traditional methods as suggested by [39].

Despite the apparent lack of *Bacillus* spp. proliferation, the oral administration of *Bacillus* spores did seem to have an effect. The consistently lower richness of the communities of the distal colon of PRO piglets on day 28 suggests that the normal colonisation pattern during nursing was hindered by the spores. This was especially evident as the increased colonic and faecal diversity in AB piglets seemed to be counteracted by spore supplementation. *Bacillus* spores have previously been reported to be able to adhere to intestinal cells [40], and it could thus be speculated that the spores were able to competitively halt the colonisation pattern seen after gentamicin administration. Previous reports on the effect of different probiotics on the microbial diversity are contradicting. In agreement with our findings, [41] found *Lactobacillus acidophilus* and *Pediococcus acidilactici* to decrease species diversity of colonic digesta from pigs in the post-weaning period. The action of decreasing the number of colonising species may have a health promoting effect when the infection pressure is high. In the current study, piglets were challenged only during the natural process of weaning, and under these circumstances, spore administration did not improve piglet health. In fact, piglets administered *Bacillus* spores tended to have a higher frequency of diarrhoea before and after weaning. As there are no general guidelines on probiotic use in pigs, the dose and interval may have been sub-optimal for use in piglets.

Weaning is a stressful event for piglets, where numerous factors contribute to physiological and microbial changes in the gut [42]. Intrinsic and extrinsic factors as litter, nursing mother, and genotype are found to have the biggest influence on the gut microbiota when the piglets still suckle, while this influence decreases after weaning [43]. As a consequence, the reduced effect of gentamicin and spore administration observed two weeks after weaning as compared to day 28 could be expected, and the event of introducing solid feed, the stress of being separated from the sow, and transported to a new environment, all seemed to have a stronger influence on the gut microbiota.

The establishment of a gut microbiota is a prerequisite for an optimal development of the intestinal immune system during early life [3], and administering antibiotics to piglets shortly after birth, has been shown to alter the expression of genes related to immunological functions [6, 44]. The genes investigated in the present study encoded pro- and inflammatory cytokines and cell-to-cell adhesion proteins; however, neither early gentamicin nor continuous spore administration was able to alter the expression of these genes. Hence, treatment apparently did not have any effect on the intestinal barrier or inflammatory status. These results may have been different if the piglets had been exposed to an infection challenge.

Age is an important driver of gut microbiota maturation and is an important influencing factor [45]. In the present study, sequencing data from day 3 and 28 clearly showed the effect of age. The microbial community on day 42 was furthermore clearly different from that on day 3 and 28. The event of weaning on day 28 causing changes in diet, environment, and stress complicates the interpretation of the gut microbial changes from 28 to 42 days of age, though. Microbial diversity of the colonic and faecal communities increased up until day 42, while it was more or less constant in the ileum. Thus the ileal community is quickly colonised by the adult-like number and proportion of species, albeit the colonising species change with age.

In conclusion, administering gentamicin to piglets on three consecutive days from 4 days of life caused an increased diversity of the colonic and faecal microbiota, which was evident 3 weeks later. Administration of *Bacillus* spores, on the other hand, decreased species diversity and hence had the opposite effect to that of gentamicin. We were not able to show that spore administration beneficially affected the health of newly weaned piglets. Yet, the importance of these findings in relation to the clinical status of the animals needs further investigation, as administration of probiotics may be beneficial to animal health and decrease the use of antibiotics, which is a necessity in combating the rise in antibiotic resistance. The study furthermore underlines the importance of distinguishing between different classes of antibiotics when discussing the impact of antibiotic treatment on dysbiosis.

## Supporting information

S1 Fig Culture of selected bacterial groups. Enumeration of selected bacterial groups (A-E) in digesta (log cfu/g) from the stomach, ileum, caecum, and mid colon sampled day 3, 28, and 42 from piglets administered gentamicin (AB; n = 71); piglets administered *Bacillus* spores (PRO; n = 68); piglets administered both gentamicin and *Bacillus* spores (PRO+AB; n = 71), and control piglets not receiving gentamicin or *Bacillus* spores (CONTROL; n = 72). Values are presented as least-square means and the 95% confidence intervals presented as vertical bars. Bars embraced by horisontal brackets market by * (0.01≤*p*<0.05), ** (0.001≤*p*<0.01) or *** (*p*<0.001) are significantly different.
(PDF)

S1 Table pH of digesta. pH of digesta from the gastrointestinal tract of 3, 28 and 48 days old piglets.
(DOCX)

S2 Table Biogenic amines in digesta. Biogenic amines (mg/kg sample) in ileum and mid colon digesta from piglets 3, 28, and 42 days of age.
(DOCX)

S3 Table Organic acids in digesta. Organic acids (mmol/kg sample) in ileum and mid colon digesta from piglets 3, 28, and 42 days of age^.^
(DOCX)

S2 Fig Principal Component Analysis; faecal samples coloured according to treatment. Principal Component Analysis (PCA) of square root transformed OTU abundances originating from faecal samples (n = 207) displaying PC1 and PC2. (A) Day 7 samples; (B) Day 28 samples; (C) Constrained (for treatment group) PCA on day 28 samples; (D) Day 42 samples; (E) Constrained (for treatment group; RDA) PCA on day 42 samples. Points are coloured according to treatment. AB: Piglets administered gentamicin; PRO: Piglets administered *Bacillus* spores; PRO+AB: Both administered gentamicin and *Bacillus* spores; CONTROL: Control piglets not receiving gentamicin or *Bacillus* spores.
(PDF)

S3 Fig Principal Component Analysis; faecal samples coloured according to age. Principal Component Analysis of square root transformed OTU abundances in faeces (n = 207) displaying PC1 and PC2. Points are coloured according to age.
(PDF)

S4 Fig Principal Component Analysis; digesta samples coloured according to treatment and grouped according to age. (A) Principal Component Analysis (PCA) of square root transformed OTU abundances in colonic digesta from day 28 (n = 17) displaying PC1 and PC2. Points are coloured according to treatment and grouped according to age. (B) Constrained PCA of square root transformed OTU abundances in colonic digesta day 28 (n = 17). Points are coloured for treatment. AB: Piglets administered gentamicin; PRO: Piglets administered *Bacillus* spores; PRO+AB: Both administered gentamicin and *Bacillus* spores; CONTROL: Control piglets not receiving gentamicin or *Bacillus* spores.
(PDF)

S5 Fig Principal Component Analysis; digesta samples coloured according to age and grouped according to intestinal segment. Principal Component Analysis of square root transformed OTU abundances in ileal (n = 61) and colonic (n = 65) digesta displaying PC1 and PC2. Points are coloured according to age and shaped according to segment.
(PDF)

S6 Fig Gene expression levels. Gene expression levels of (A) TNF-α, (B) IL-10, (C) Cyclo-oxygenase-2, (D) ZO-1, (E) OCLN, (F) CLDN-4, and (G) CLDN-2 in ileal tissue collected day 28 (n = 22) and 42 (n = 34) from piglets administered gentamicin (AB); piglets administered *Bacillus* spores (PRO); piglets administered both gentamicin and *Bacillus* spores (PRO+AB); and control piglets not receiving gentamicin or *Bacillus* spores (CONTROL), that have either been left untreated or stimulated with LPS. Values are presented as least-square means and the 95% confidence intervals presented as vertical bars. Bars embraced by horisontal brackets market by * (0.01≤*p*<0.05), ** (0.001≤*p*<0.01) or *** (*p*<0.001) are significantly different.
(PDF)
