# Supplementary material for: Impact of Bacillus spp. spores and gentamicin on the gastrointestinal microbiota of suckling and newly weaned piglets
Source: PLoS One. 2018 Nov 27;13(11):e0207382. doi: 10.1371/journal.pone.0207382 (PMC6258502; doi:10.1371/journal.pone.0207382)
Supplement: S1 Fig — Enumeration of selected bacterial groups (A-E) in digesta (log cfu/g) from the stomach, ileum, caecum, and mid colon sampled day 3, 28, and 42 from piglets administered gentamicin (AB; n = 71); piglets administered Bacillus spores (PRO; n = 68); piglets administered both gentamicin and Bacillus spores (PRO+AB; n = 71), and control piglets not receiving gentamicin or Bacillus spores (CONTROL; n = 72). Values are presented as least-square means and the 95% confidence intervals presented as vertical bars. Bars embraced by horisontal brackets market by * (0.01≤p<0.05), ** (0.001≤p<0.01) or *** (p<0.001) are significantly different. (PDF) [file pone.0207382.s001.pdf]

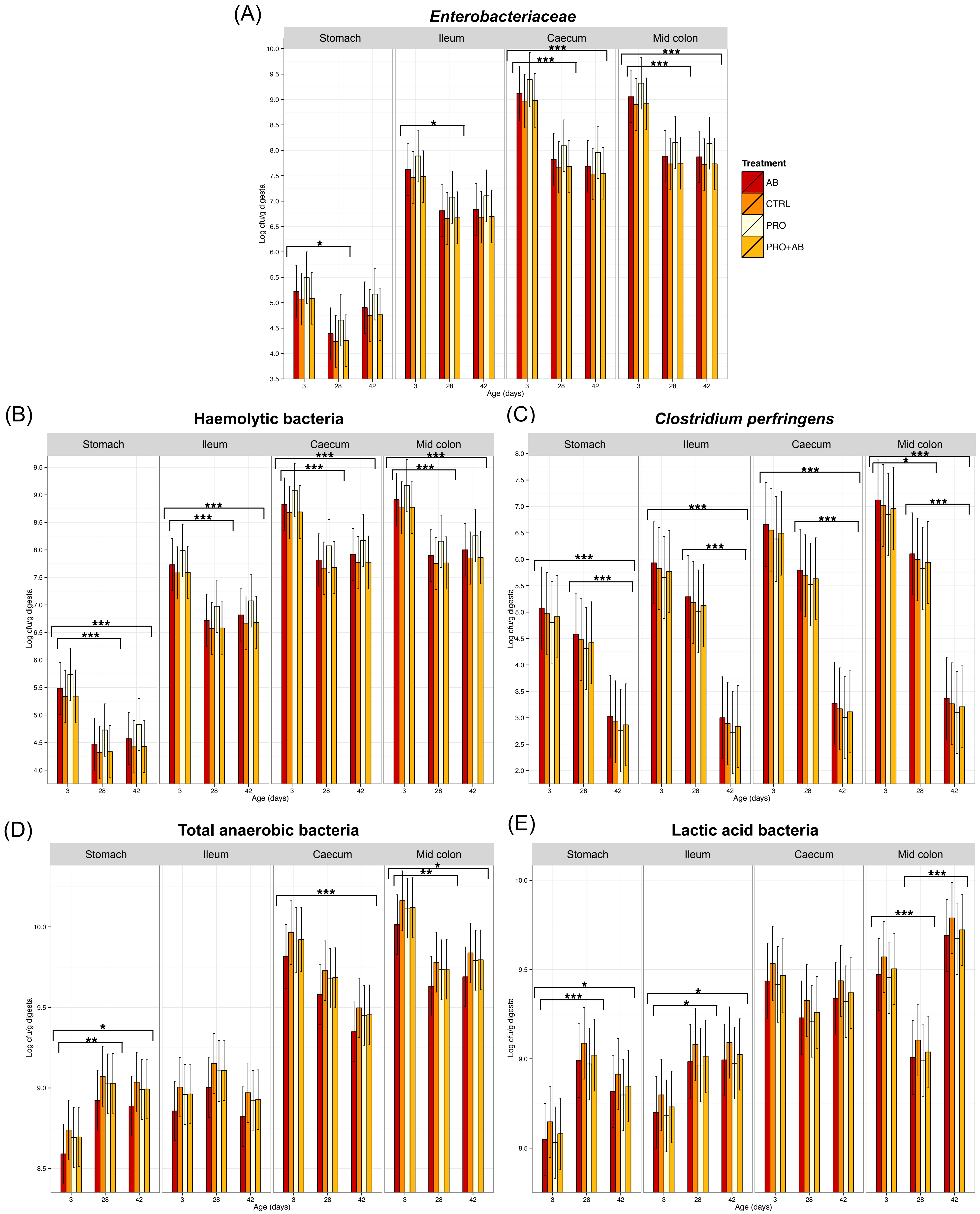
(A)
Enterobacteriaceae
(B)
Haemolytic bacteria
(C)
Clostridium perfringens
(D)
Total anaerobic bacteria
(E)
Lactic acid bacteria
Stomach
Ileum
Caecum
Mid colon
Log cfu/g digesta
Age (days)
Treatment
AB
CTRL
PRO
PRO+AB
