# Supplementary material for: Impact of Bacillus spp. spores and gentamicin on the gastrointestinal microbiota of suckling and newly weaned piglets
Source: PLoS One. 2018 Nov 27;13(11):e0207382. doi: 10.1371/journal.pone.0207382 (PMC6258502; doi:10.1371/journal.pone.0207382)
Supplement: S1 Table — pH of digesta from the gastrointestinal tract of 3, 28 and 48 days old piglets. (DOCX) [file pone.0207382.s002.docx]

**Additional file 2.** pH of digesta^1^

|  | Treatment group^2^ | | | | | | | | *p*-value |  |
| --- | --- | --- | --- | --- | --- | --- | --- | --- | --- | --- |
|  | CTRL | | AB | | PRO | | PRO+AB | | T^3^ | S*A^4^ |
| *pH* |  | |  | |  | |  | | 0.17 | <0.001 |
| Day 3 |  |  |  |  |  |  |  |  |  |  |
| Stomach | 3.5 | (3.3-3.7) | 3.7 | (3.4-3.9) | 3.7 | (3.4-3.9) | 3.6 | (3.4-3.8) |  |  |
| Ileum | 6.6 | (6.4-6.8) | 6.8 | (6.6-7.0) | 6.8 | (6.6-7.0) | 6.7 | (6.5-6.9) |  |  |
| Caecum | 6.7 | (6.4-6.9) | 6.9 | (6.6-7.1) | 6.9 | (6.6-7.2) | 6.8 | (6.6-7.1) |  |  |
| Mid colon | 6.7 | (6.5-6.9) | 6.9 | (6.6-7.1) | 6.9 | (6.6-7.1) | 6.8 | (6.6-7.0) |  |  |
| Day 28 |  |  |  |  |  |  |  |  |  |  |
| Stomach | 3.7 | (3.5-3.9) | 3.9 | (3.7-4.1) | 3.9 | (3.7-4.1) | 3.8 | (3.6-4.1) |  |  |
| Ileum | 6.7 | (6.5-6.9) | 6.9 | (6.7-7.1) | 6.9 | (6.7-7.1) | 6.8 | (6.6-7.1) |  |  |
| Caecum | 6.1 | (5.9-6.3) | 6.3 | (6.1-6.5) | 6.3 | (6.1-6.5) | 6.3 | (6.0-6.5) |  |  |
| Mid colon | 6.4 | (6.2-6.6) | 6.6 | (6.4-6.8) | 6.6 | (6.4-6.8) | 6.5 | (6.3-6.8) |  |  |
| Day 42 |  |  |  |  |  |  |  |  |  |  |
| Stomach | 3.2 | (3.0-3.4) | 3.4 | (3.2-3.6) | 3.4 | (3.2-3.6) | 3.3 | (3.1-3.6) |  |  |
| Ileum | 6.5 | (6.3-6.7) | 6.7 | (6.5-6.9) | 6.7 | (6.5-6.9) | 6.6 | (6.4-6.8) |  |  |
| Caecum | 5.9 | (5.6-6.1) | 6.0 | (5.8-6.3) | 6.1 | (5.8-6.3) | 6.0 | (5.8-6.2) |  |  |
| Mid colon | 6.3 | (6.1-6.5) | 6.5 | (6.2-6.7) | 6.5 | (6.3-6.7) | 6.4 | (6.2-6.6) |  |  |

pH of digesta from the gastrointestinal tract of 3, 28 and 48 days old piglets.

^1^ Values are presented as least square means and 95% confidence intervals (in parentheses).

^2^ CTRL = control; AB = antibiotic group; PRO = probiotic group; PRO+AB = probiotic+antibiotic group. Number of piglets: CTRL= 32; AB=32; PRO=31; PRO+AB= 32.

^3^ Treatment group.

^4^ S*A = interaction between intestinal segment and age.
