# Supplementary material for: Impact of Bacillus spp. spores and gentamicin on the gastrointestinal microbiota of suckling and newly weaned piglets
Source: PLoS One. 2018 Nov 27;13(11):e0207382. doi: 10.1371/journal.pone.0207382 (PMC6258502; doi:10.1371/journal.pone.0207382)
Supplement: S2 Table — Biogenic amines (mg/kg sample) in ileum and mid colon digesta from piglets 3, 28, and 42 days of age. (DOCX) [file pone.0207382.s003.docx]

**Additional file 3.** Biogenic amines in digesta.

| Treatment group^2^ | | | | | | | | | |  | *p*-value | |  |  |  |  |
| --- | --- | --- | --- | --- | --- | --- | --- | --- | --- | --- | --- | --- | --- | --- | --- | --- |
|  | CTRL | | AB | | | PRO | | PRO+AB | | # | T^3^ | S*A^4^ |  |  |  |  |
| Cadaverine | | | | | | | | | |  | 0.39 | <0.001 |  |  |  |  |
| Day 3 | | | | | | | | | | | |  |  |  |  |  |
| Ileum | 5.4 | (0.1-18.3) | 2.4 | (0-11.4) | | 9.0 | (1.5-27.0) | 4.6 | (0-16.3) | a |  |  |  |  |  |  |
| Mid colon | 205.7 | (83.0-502.5) | 136.6 | (54.3-335.6) | | 287.0 | (116.4-700.1) | 186.3 | (74.9-455.4) | A |  |  |  |  |  |  |
| Day 28 |  |  |  |  | |  |  |  |  |  |  |  |  |  |  |  |
| Ileum | 9.6 | (2.4-25.5) | 5.2 | (0.4-16.2) | | 14.8 | (4.6-37.5) | 8.4 | (1.8-22.8) | a |  |  |  |  |  |  |
| Mid colon | 24.2 | (9.1-57.1) | 15.0 | (4.9-37.3) | | 35.0 | (14.0-80.9) | 21.6 | (7.9-51.4) | B |  |  |  |  |  |  |
| Day 42 | | | | | | | | | | | |  |  |  |  |  |
| Ileum | 115.5 | (51.0-256.1) | 76.1 | | (32.9-170.6) | 161.8 | (72.0-357.8) | 104.4 | (45.9-231.9) | b |  |  |  |  |  |  |
| Mid colon | 70.3 | (30.2-157.5) | 45.8 | | (19.0-104.6) | 99.0 | (43.3-220.7) | 63.4 | (27.1-142.5) | AB |  |  |  |  |  |  |
| Putrescine | | | | | | | | | |  | 0.28 | <0.001 |  |  |  |  |
| Day 3 | | | | | | | | | | | |  |  |  |  |  |
| Ileum | 19.2 | (7.6-43.3) | 9.6 | | (2.9-23.2) | 22.3 | (9.0-49.6) | 15.7 | (5.9-36.0) | a |  |  |  |  |  |  |
| Mid colon | 526.9 | (253.8-1089.9) | 302.3 | | (144.9-626.5) | 598.2 | (288.1-1238.5) | 444.7 | (213.9-920.4) | A |  |  |  |  |  |  |
| Day 28 | | | | | | | | | | | |  |  |  |  | Day 28 |
| Ileum | 21.1 | (9.4-43.4) | 10.7 | | (4.0-23.3) | 24.4 | (11.0-50.4) | 17.3 | (7.4-36.2) | a |  |  |  |  |  |  |
| Mid colon | 18.9 | (8.3-39.1) | 9.5 | | (3.4-20.8) | 21.9 | (9.9-44.9) | 15.5 | (6.5-32.5) | B |  |  |  |  |  |  |
| Day 42 | | | | | | | | | | | |  |  |  |  | Day 42 |
| Ileum | 60.1 | (30.0-117.4) | 33.2 | | (15.9-66) | 68.7 | (34.4-134.0) | 50.3 | (24.8-98.7) | b |  |  |  |  |  |  |
| Mid colon | 44.8 | (21.9-88.2) | 24.4 | | (11.2-49.1) | 51.2 | (25.3-100.7) | 37.3 | (18.0-74.0) | B |  |  |  |  |  |  |
|  |  |  |  | |  |  |  |  |  |  |  |  |  |  |  |  |

Biogenic amines^1^ (mg/kg sample) in ileum and mid colon digesta from piglets 3, 28, and 42 days of age_._

^1^ Values are presented as least square means and 95% confidence intervals (in parentheses).

^2^ CTRL = control; AB = antibiotic group; PRO = probiotic group; PRO+AB = probiotic+antibiotic group.

Number of piglets: CTRL=32; AB=32; PRO=31; PRO+AB=32.

^3^ T = treatment group

^4^ S*A = interaction between intestinal segment and age.

a, b: Ileum samples. Rows with different letters, within a biogenic amine group, are significantly different (*p*<0.05).

A, B: Mid colon samples. Rows with different letters, within a biogenic amine group, are significantly different (*p*<0.05).

Most agmatine and tyramine measurements were below detection level.
