# Supplementary material for: Impact of Bacillus spp. spores and gentamicin on the gastrointestinal microbiota of suckling and newly weaned piglets
Source: PLoS One. 2018 Nov 27;13(11):e0207382. doi: 10.1371/journal.pone.0207382 (PMC6258502; doi:10.1371/journal.pone.0207382)
Supplement: S3 Table — Organic acids (mmol/kg sample) in ileum and mid colon digesta from piglets 3, 28, and 42 days of age. (DOCX) [file pone.0207382.s004.docx]

**Additional file 4.** Organic acids in digesta.

| Treatment group^2^ | | | | | | | | |  | *p*-value |  |
| --- | --- | --- | --- | --- | --- | --- | --- | --- | --- | --- | --- |
|  | CTRL | | AB | | PRO | | PRO+AB | | # | T^3^ | S*A^4^ |
| Acetic acid |  |  |  |  |  |  |  |  |  | 0.55 | <0.001 |
| Day 3 |  |  |  |  |  |  |  |  |  |  |  |
| Ileum | 0.9 | (0.6-3.9) | 1.0 | (0.6-4.4) | 0.8 | (0.5-3.4) | 0.8 | (0.5-3.0) | a |  |  |
| Mid colon | 4.1 | (2.6-21.8) | 4.5 | (2.9-24.2) | 3.7 | (2.4-19.7) | 3.4 | (2.2-17.8) | A |  |  |
| Day 28 |  |  |  |  |  |  |  |  |  |  |  |
| Ileum | 1.5 | (1.0-6.9) | 1.7 | (1.1-7.7) | 1.4 | (0.9-6.2) | 1.3 | (0.8-5.5) | ab |  |  |
| Mid colon | 5.7 | (3.9-29.7) | 6.4 | (4.3-33.0) | 5.2 | (3.6-27.0) | 4.8 | (3.2-24.5) | A |  |  |
| Day 42 |  |  |  |  |  |  |  |  |  |  |  |
| Ileum | 1.9 | (1.3-8.8) | 2.1 | (1.4-9.9) | 1.7 | (1.2-7.9) | 1.5 | (1.1-7.1) | b |  |  |
| Mid colon | 20.6 | (14.0-110.1) | 22.8 | (15.5-121.9) | 18.8 | (12.7-100.3) | 17.1 | (11.6-91.1) | B |  |  |
| Propionic acid |  |  |  |  |  |  |  |  |  | 0.56 | <0.001 |
| Day 3 |  |  |  |  |  |  |  |  |  |  |  |
| Ileum | 0.4 | (0.4-0.7) | 0.4 | (0.4-0.9) | 0.4 | (0.4-0.7) | 0.4 | (0.4-0.5) |  |  |  |
| Mid colon | 3.3 | (2.0-5.2) | 3.9 | (2.4-6.2) | 3.5 | (2.2-5.6) | 2.9 | (1.7-4.6) | A |  |  |
| Day 28 |  |  |  |  |  |  |  |  |  |  |  |
| Ileum | 0.5 | (0.4-1.0) | 0.4 | (0.4-0.9) | 0.6 | (0.4-1.1) | 0.4 | (0.4-0.8) |  |  |  |
| Mid colon | 6.5 | (4.4-9.6) | 7.7 | (5.2-11.2) | 7.0 | (4.7-10.2) | 5.8 | (3.9-8.5) | B |  |  |
| Day 42 |  |  |  |  |  |  |  |  |  |  |  |
| Ileum | 0.4 | (0.4-0.5) | 0.4 | (0.4-0.9) | 0.4 | (0.4-0.5) | 0.4 | (0.4-0.4) |  |  |  |
| Mid colon | 28.5 | (19.8-40.8) | 33.3 | (23.2-47.7) | 30.2 | (21-43.4) | 25.3 | (17.6-36.4) | C |  |  |
| Butyric acid |  |  |  |  |  |  |  |  |  | 0.93 | Not tested |
| Mid colon |  |  |  |  |  |  |  |  |  |  |  |
| Day 3 | 2.7 | (0.3-5.1) | 2.0 | (0-4.4) | 2.0 | (0-4.4) | 2.1 | (0-4.4) | A |  |  |
| Day 28 | 4.3 | (2.2-6.4) | 3.6 | (1.5-5.7) | 3.6 | (1.4-5.7) | 3.6 | (1.5-5.8) | A |  |  |
| Day 42 | 11.2 | (9.1-13.3) | 10.5 | (8.4-12.6) | 10.5 | (8.4-12.6) | 10.6 | (8.4-12.7) | B |  |  |
| Valeric acid |  |  |  |  |  |  |  |  |  | 0.87 | Not tested |
| Mid colon |  |  |  |  |  |  |  |  |  |  |  |
| Day 3 | 0.9 | (0.1-1.7) | 0.5 | (0-1.3) | 0.6 | (0-1.4) | 0.6 | (0-1.3) | A |  |  |
| Day 28 | 2.0 | (1.3-2.8) | 1.7 | (1.0-2.4) | 1.8 | (1.1-2.5) | 1.7 | (1.0-2.5) | B |  |  |
| Day 42 | 2.8 | (2.1-3.5) | 2.5 | (1.7-3.2) | 2.5 | (1.8-3.3) | 2.5 | (1.8-3.2) | C |  |  |
| Iso-butyric+iso-valeric acid |  |  |  |  |  |  |  |  |  | 0.83 | Not tested |
| Mid colon |  |  |  |  |  |  |  |  |  |  |  |
| Day 3 | 1.3 | (0.8-2.1) | 1.3 | (0.8-2.2) | 1.4 | (0.8-2.3) | 1.1 | (0.7-1.8) |  |  |  |
| Day 28 | 1.8 | (1.2-2.8) | 1.9 | (1.2-3.0) | 2.0 | (1.3-3.1) | 1.6 | (1.0-2.4) |  |  |  |
| Day 42 | 2.4 | (1.5-3.7) | 2.5 | (1.6-3.9) | 2.6 | (1.6-4.0) | 2.0 | (1.3-3.2) |  |  |  |
| Lactic acid |  |  |  |  |  |  |  |  |  | 0.41 | Not tested |
| Ileum |  |  |  |  |  |  |  |  |  |  |  |
| Day 3 | 10.9 | (7.0-16.3) | 10.7 | (6.9-16.0) | 7.7 | (4.7-11.8) | 9.1 | (5.7-13.7) | A |  |  |
| Day 28 | 10.1 | (6.8-14.6) | 9.9 | (6.6-14.3) | 7.0 | (4.4-10.6) | 8.3 | (5.5-12.2) | A |  |  |
| Day 42 | 38.4 | (27.7-52.7) | 37.7 | (27.2-51.8) | 28.6 | (20.4-39.7) | 32.8 | (23.6-45.2) | B |  |  |

Organic acids^1^ (mmol/kg sample) in ileum and mid colon digesta from piglets 3, 28, and 42 days of age.

^1^ Values are presented as least square means and 95% confidence intervals (in parentheses).

^2^ CTRL = control; AB = antibiotic group; PRO = probiotic group; PRO+AB = probiotic+antibiotic group.

Number of piglets: CTRL=32; AB=32; PRO=31; PRO+AB=32.

^3^ T = treatment group.

^4^ S*A = interaction between intestinal segment and age. NB: Not tested for all parameters, as only one segment was analysed.

a, b: Ileal samples. Rows with different letters, within an acid group, are significantly different (*p*<0.05).

A, B, C: Mid colon samples. Rows with different letters, within an acid group, are significantly different (*p*<0.05).
