# Supplementary figures and images for: Impact of Bacillus spp. spores and gentamicin on the gastrointestinal microbiota of suckling and newly weaned piglets
Source: PLoS One. 2018 Nov 27;13(11):e0207382. doi: 10.1371/journal.pone.0207382 (PMC6258502; doi:10.1371/journal.pone.0207382)

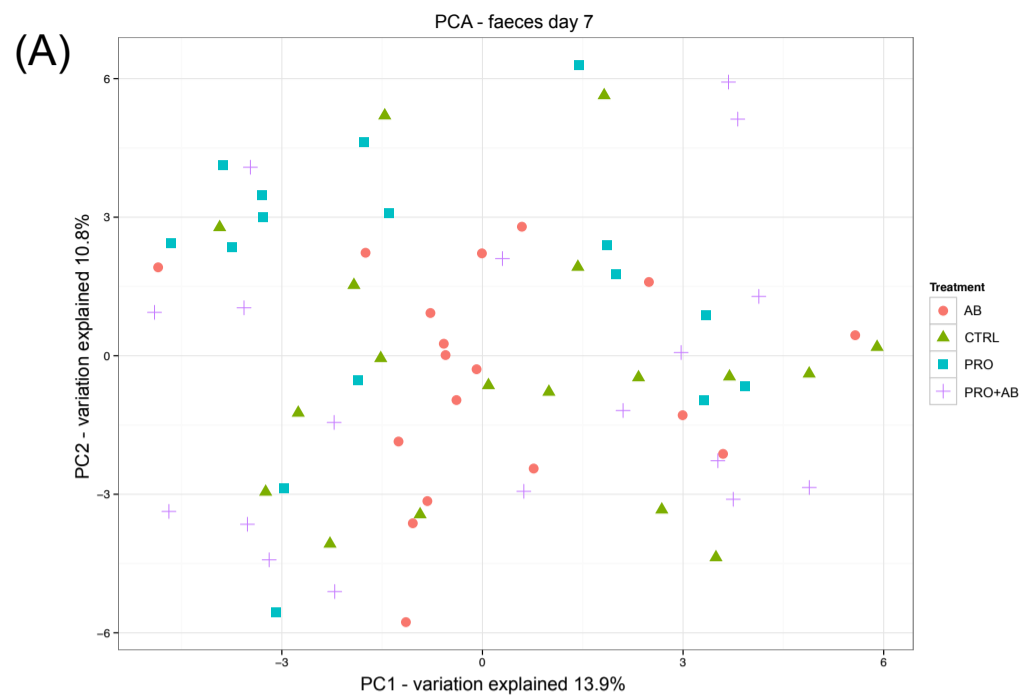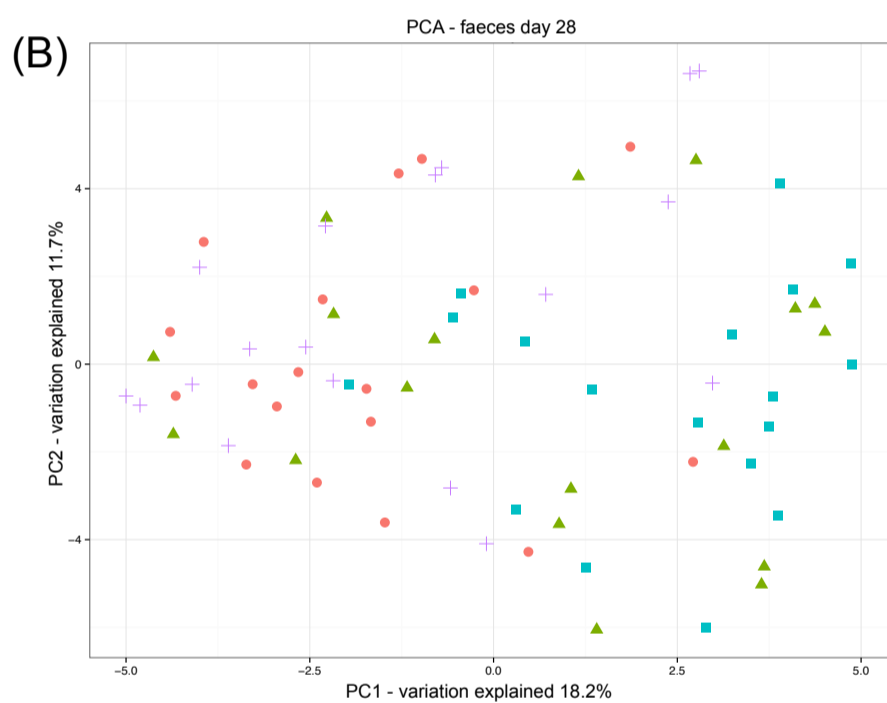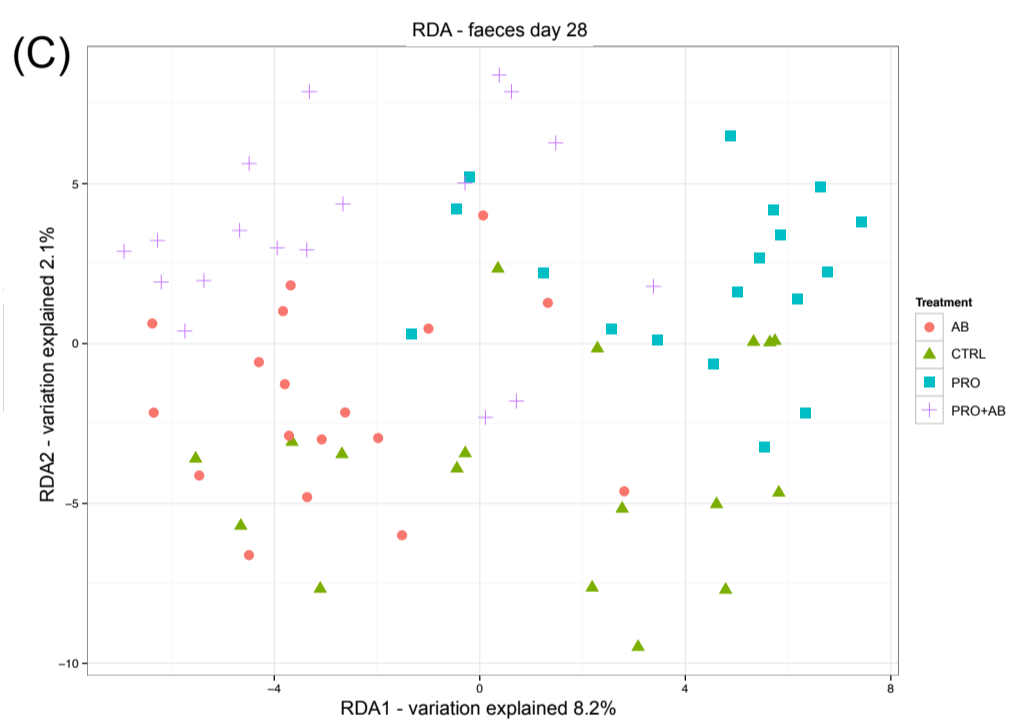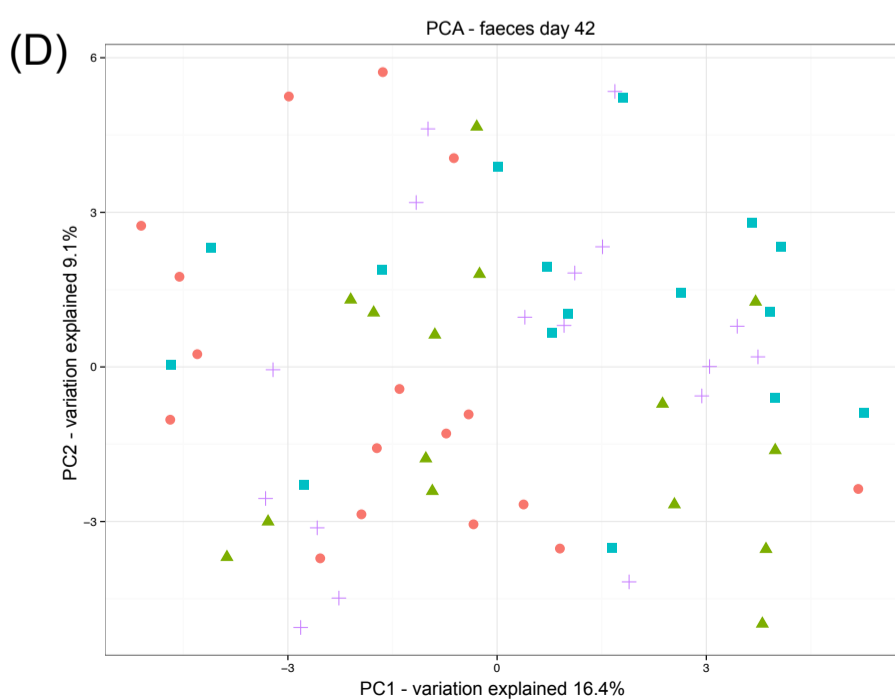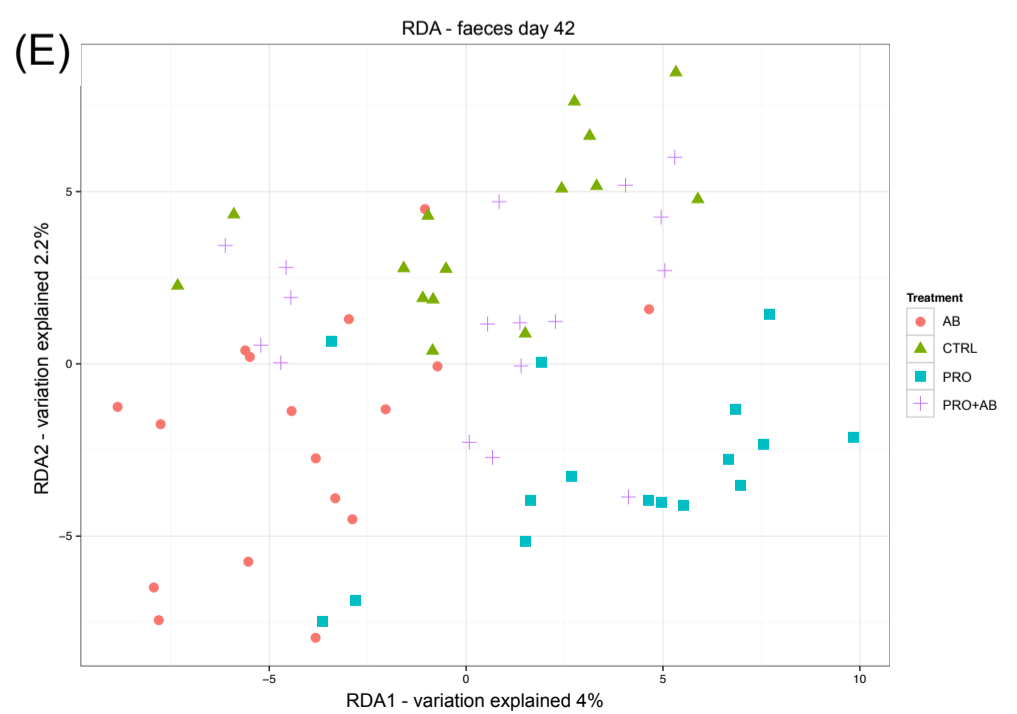

Supplement: S2 Fig — Principal Component Analysis (PCA) of square root transformed OTU abundances originating from faecal samples (n = 207) displaying PC1 and PC2. (A) Day 7 samples; (B) Day 28 samples; (C) Constrained (for treatment group) PCA on day 28 samples; (D) Day 42 samples; (E) Constrained (for treatment group; RDA) PCA on day 42 samples. Points are coloured according to treatment. AB: Piglets administered gentamicin; PRO: Piglets administered Bacillus spores; PRO+AB: Both administered gentamicin and Bacillus spores; CONTROL: Control piglets not receiving gentamicin or Bacillus spores. (PDF) [file pone.0207382.s005.pdf]

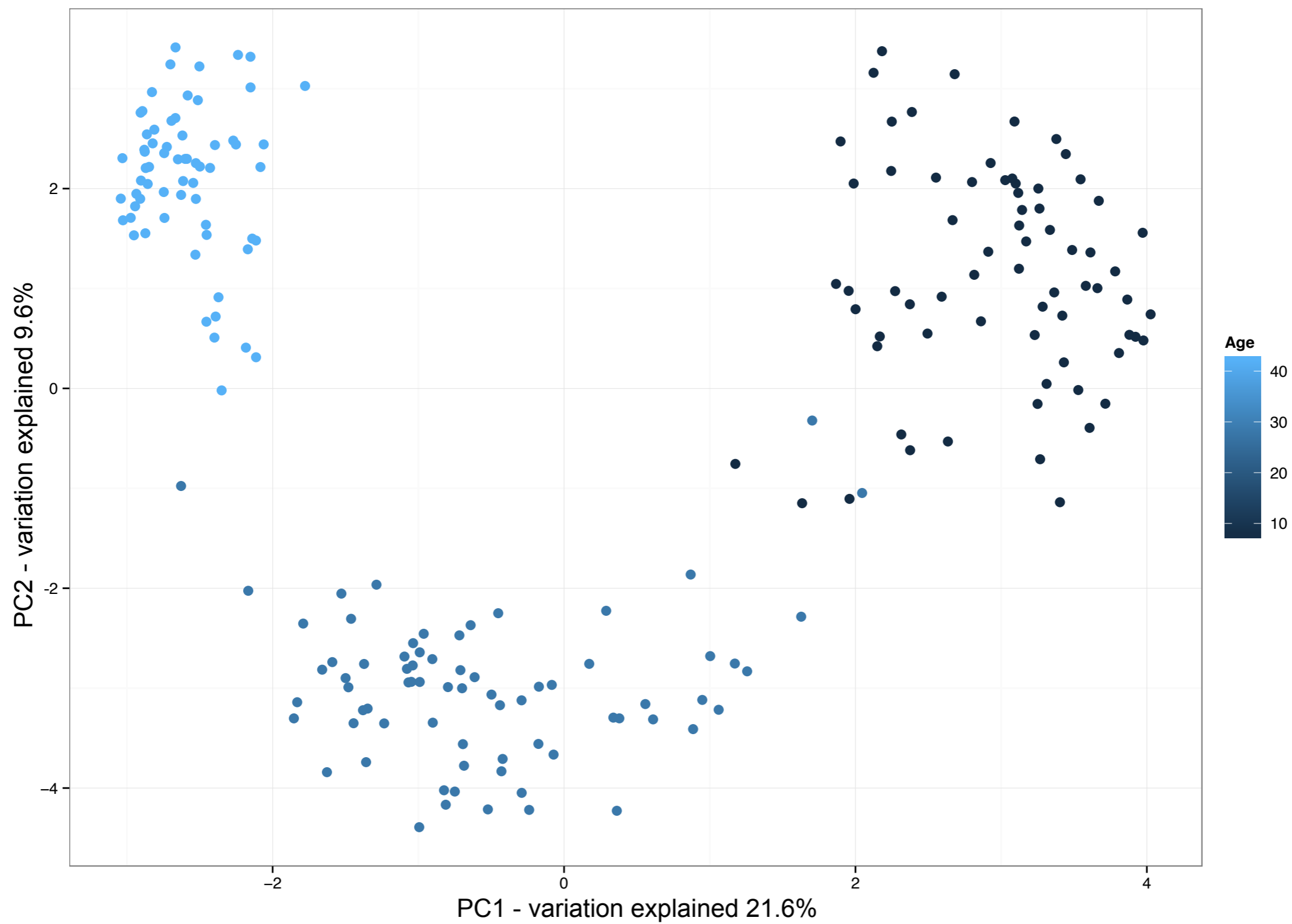

Supplement: S3 Fig — Principal Component Analysis of square root transformed OTU abundances in faeces (n = 207) displaying PC1 and PC2. Points are coloured according to age. (PDF) [file pone.0207382.s006.pdf]

(A)

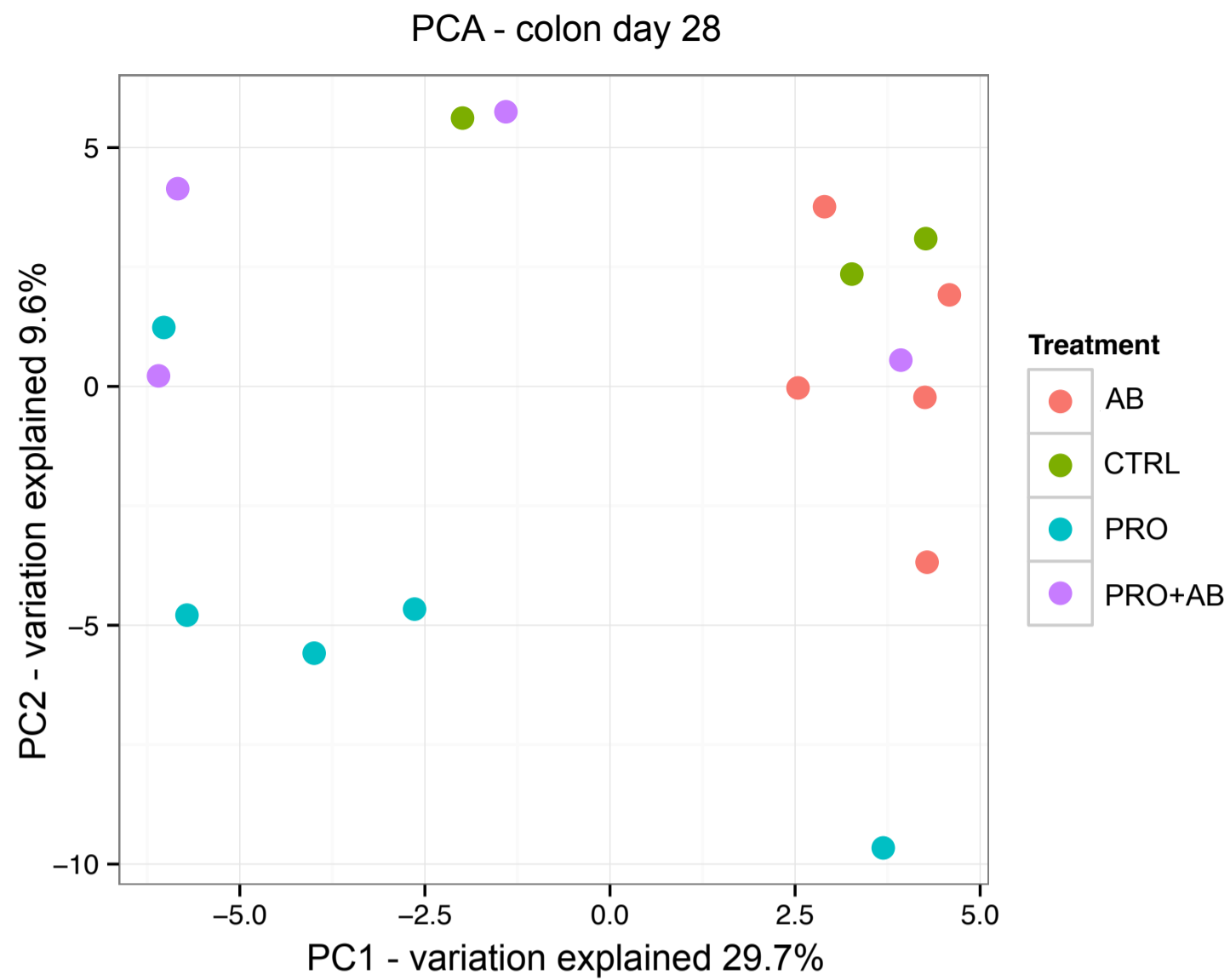

(B)

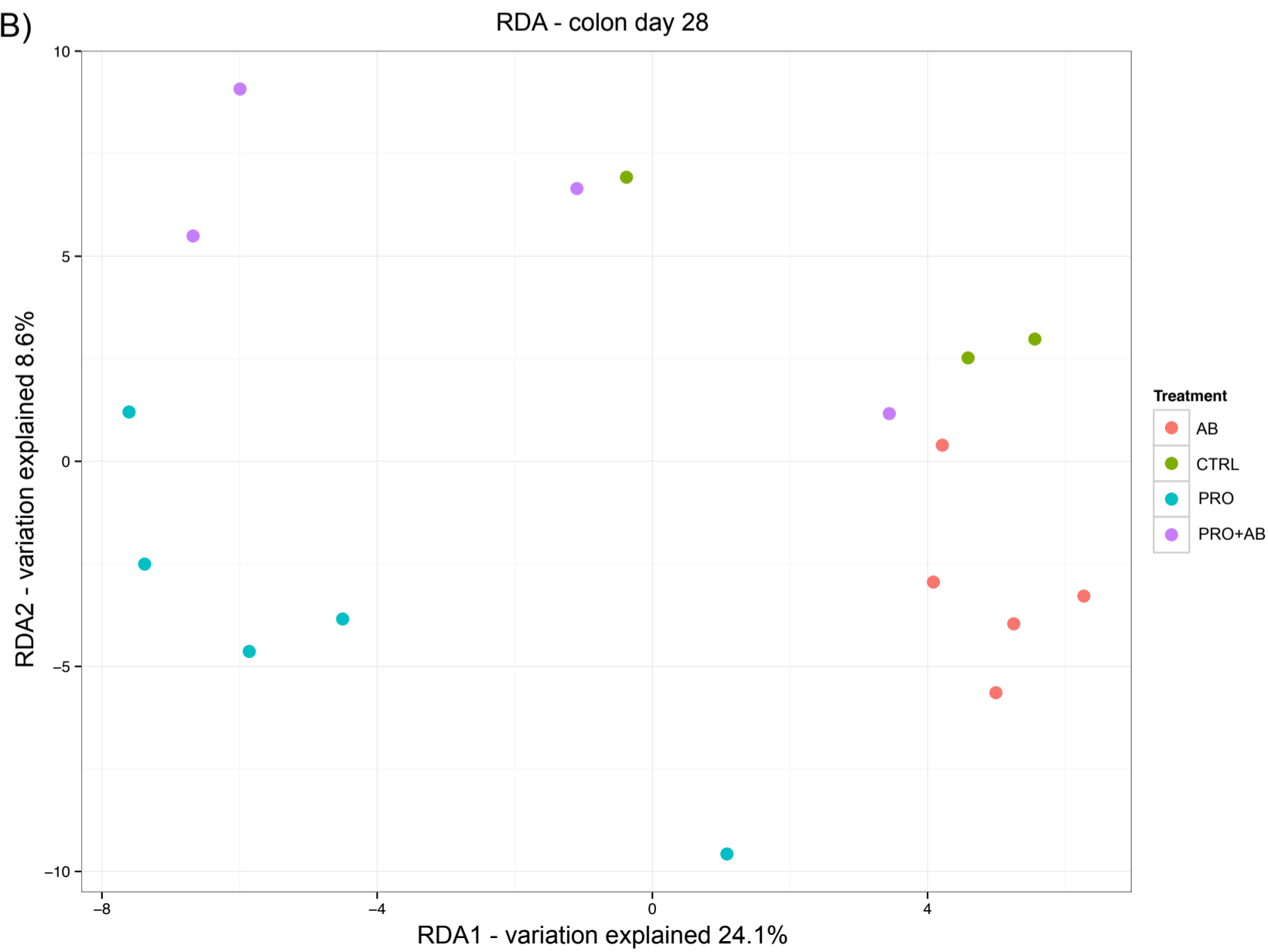

Supplement: S4 Fig — (A) Principal Component Analysis (PCA) of square root transformed OTU abundances in colonic digesta from day 28 (n = 17) displaying PC1 and PC2. Points are coloured according to treatment and grouped according to age. (B) Constrained PCA of square root transformed OTU abundances in colonic digesta day 28 (n = 17). Points are coloured for treatment. AB: Piglets administered gentamicin; PRO: Piglets administered Bacillus spores; PRO+AB: Both administered gentamicin and Bacillus spores; CONTROL: Control piglets not receiving gentamicin or Bacillus spores. (PDF) [file pone.0207382.s007.pdf]

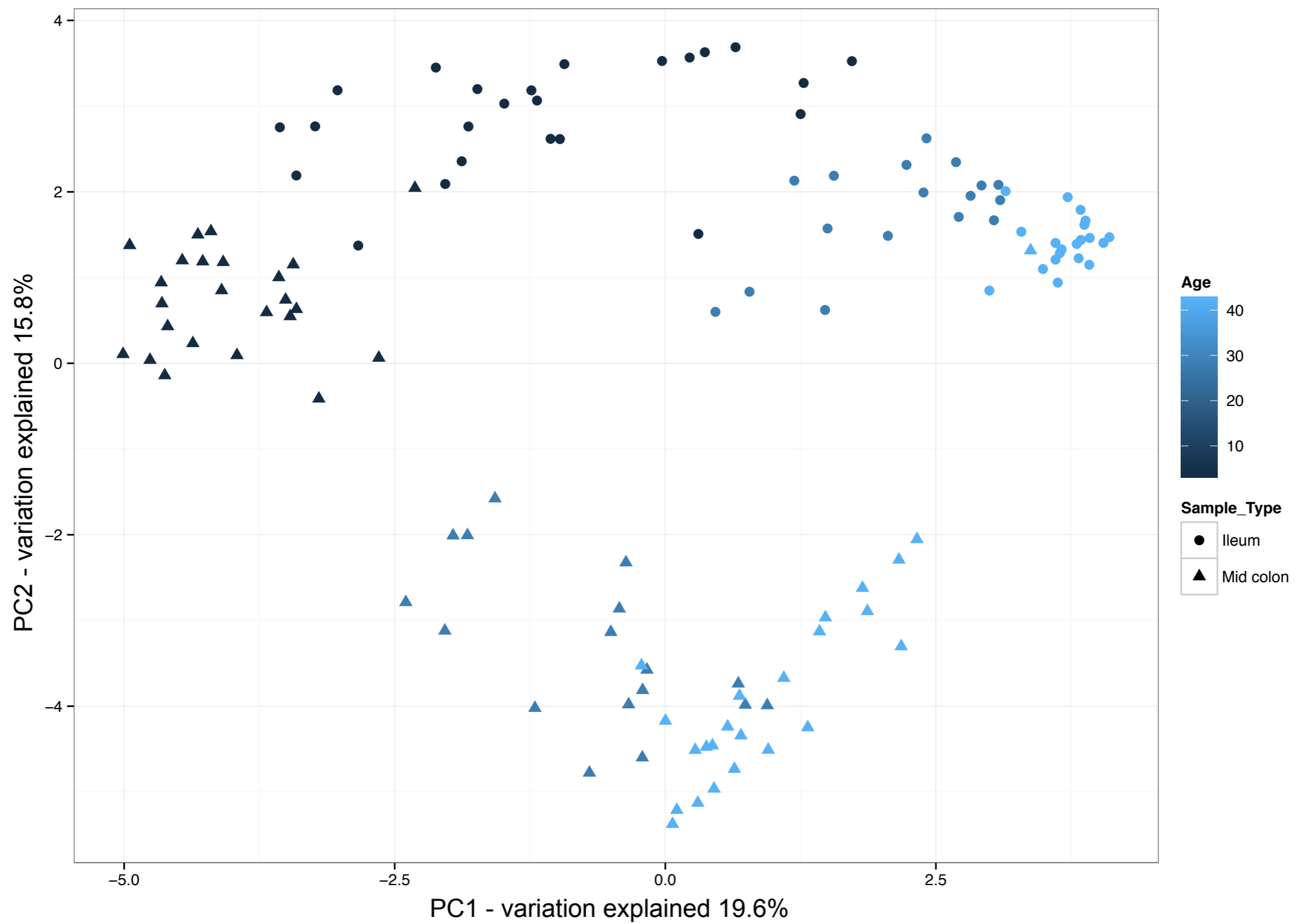

Supplement: S5 Fig — Principal Component Analysis of square root transformed OTU abundances in ileal (n = 61) and colonic (n = 65) digesta displaying PC1 and PC2. Points are coloured according to age and shaped according to segment. (PDF) [file pone.0207382.s008.pdf]

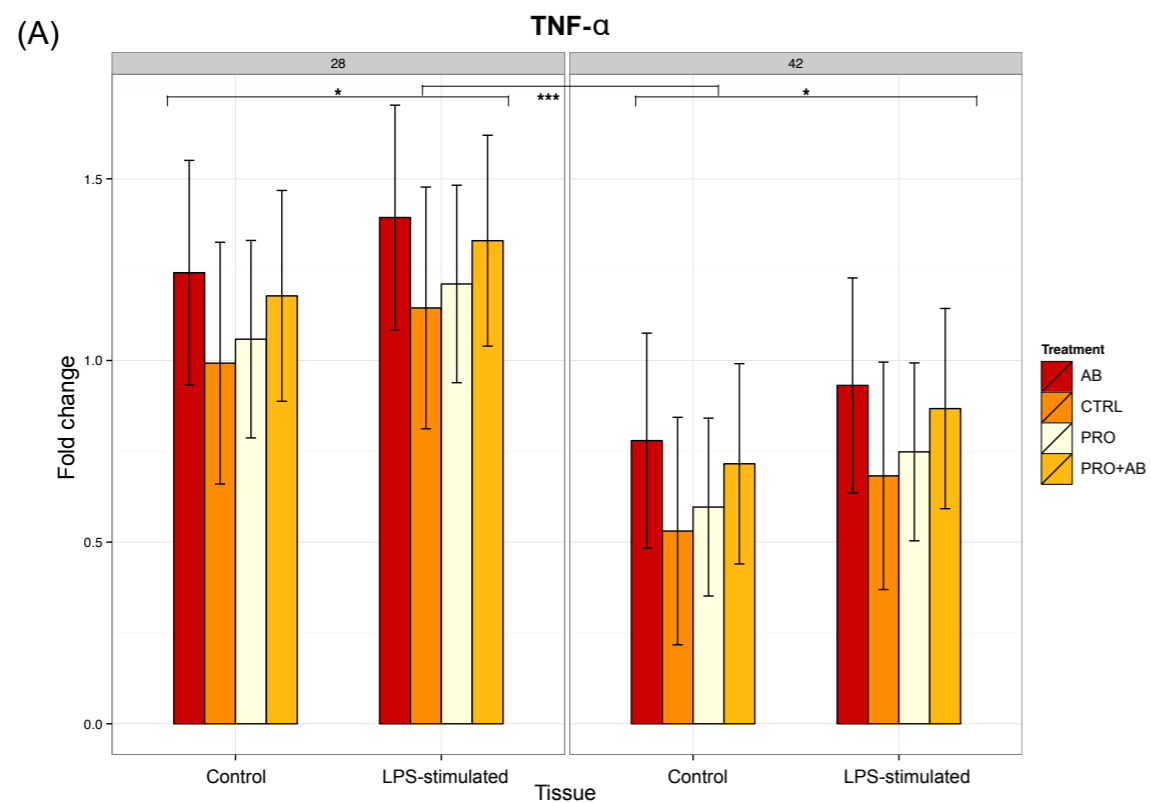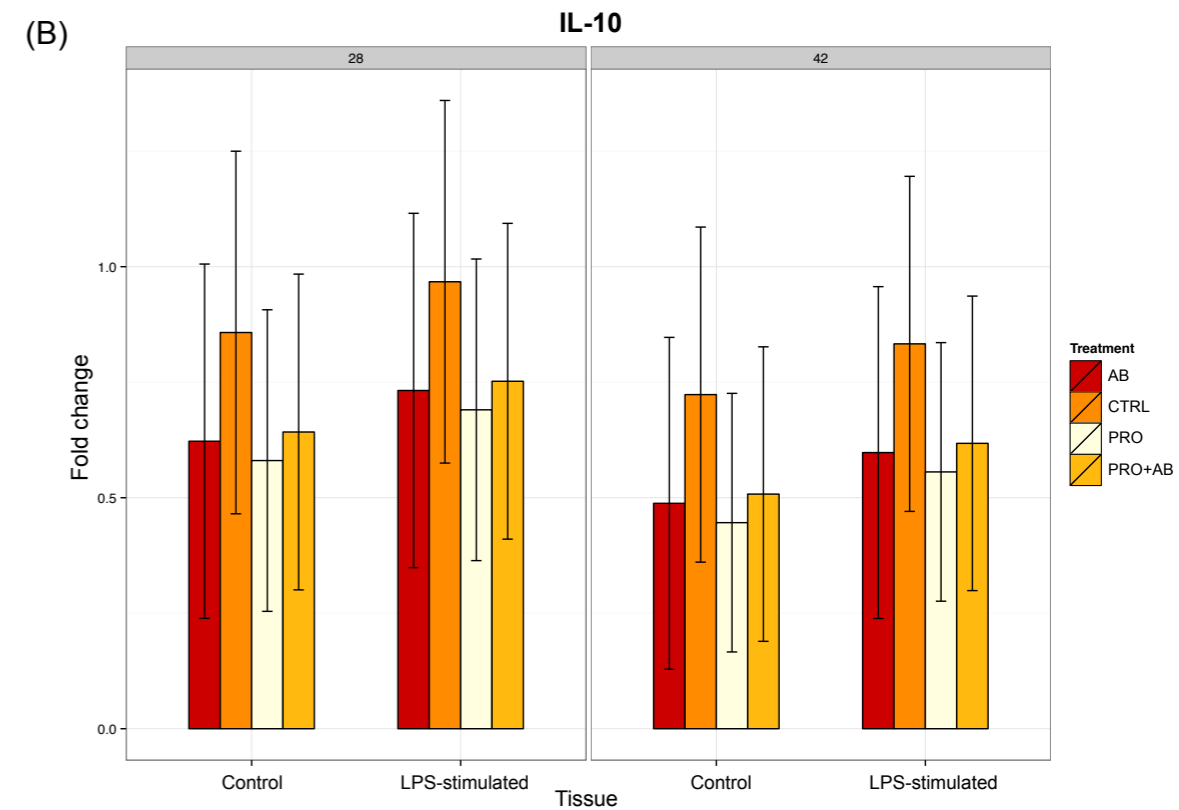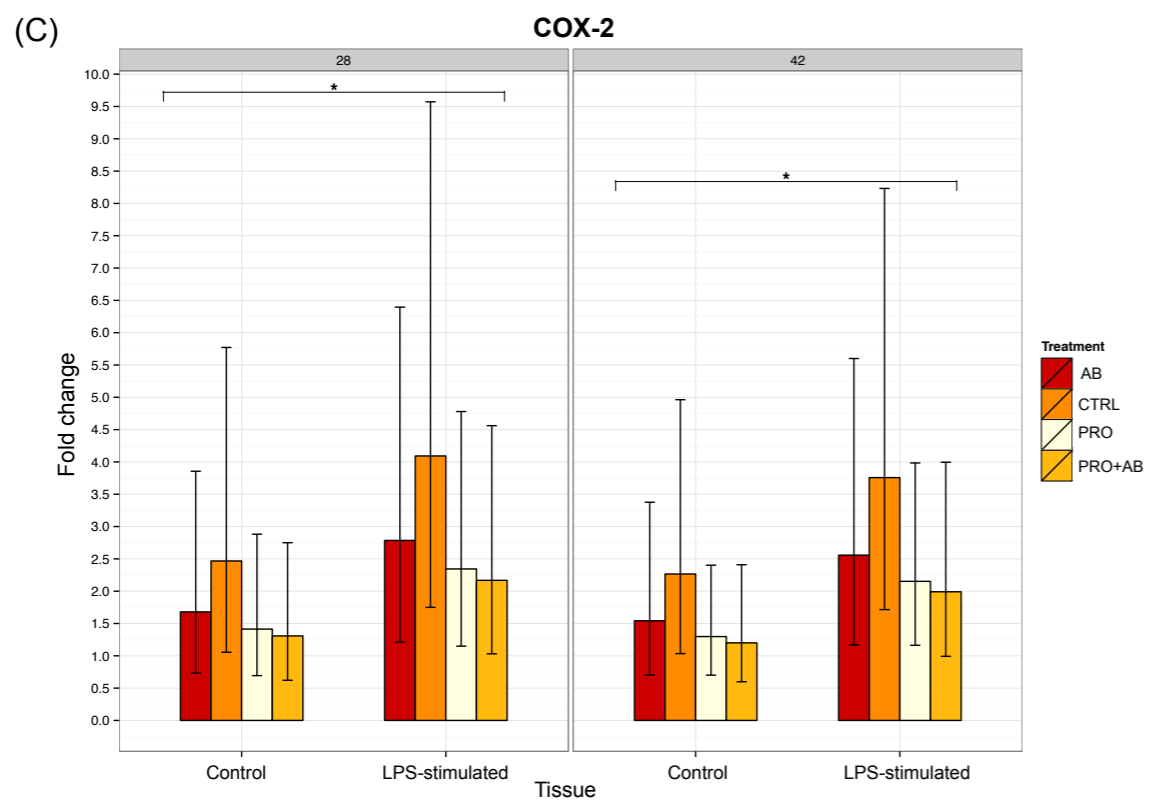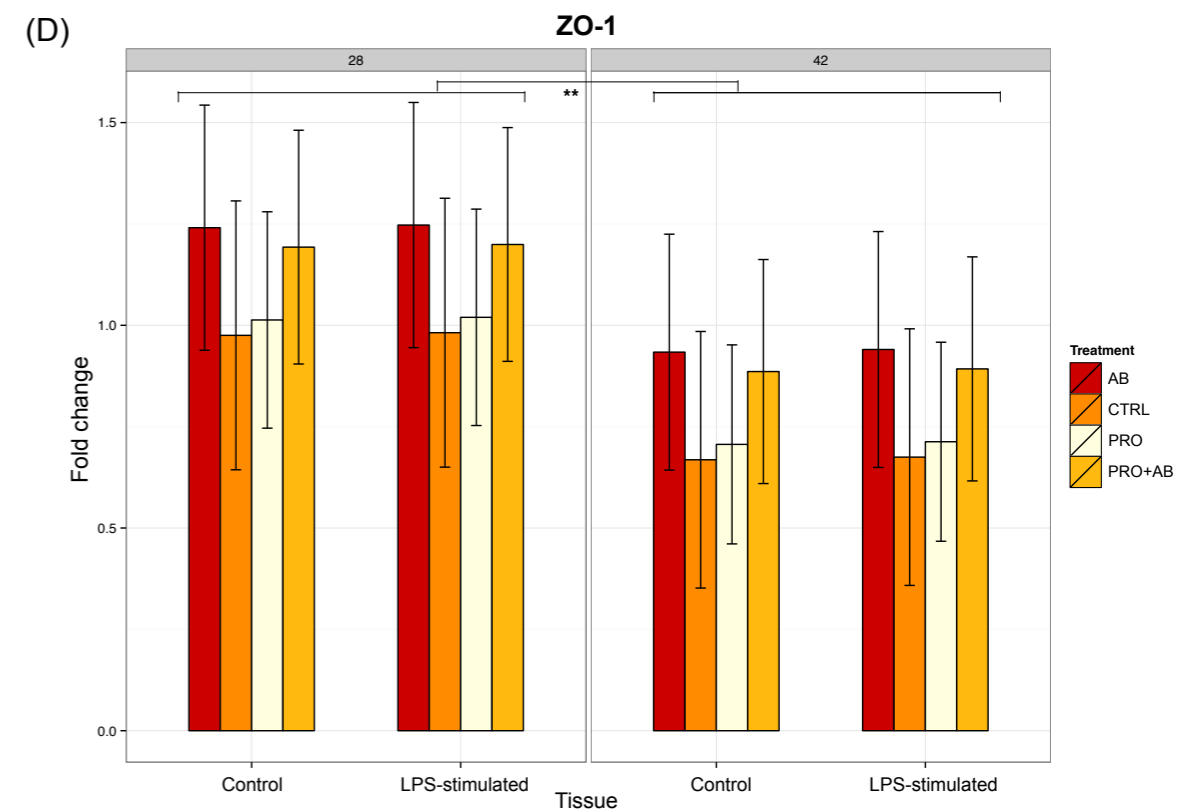

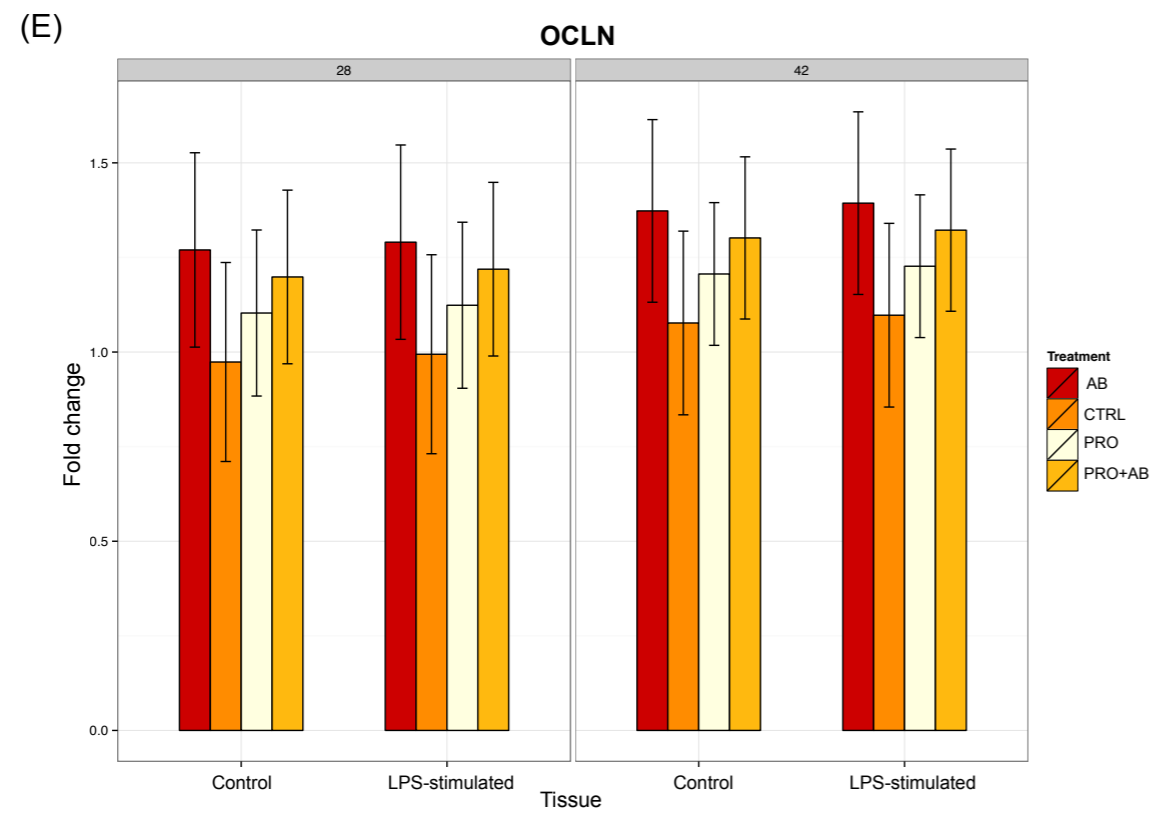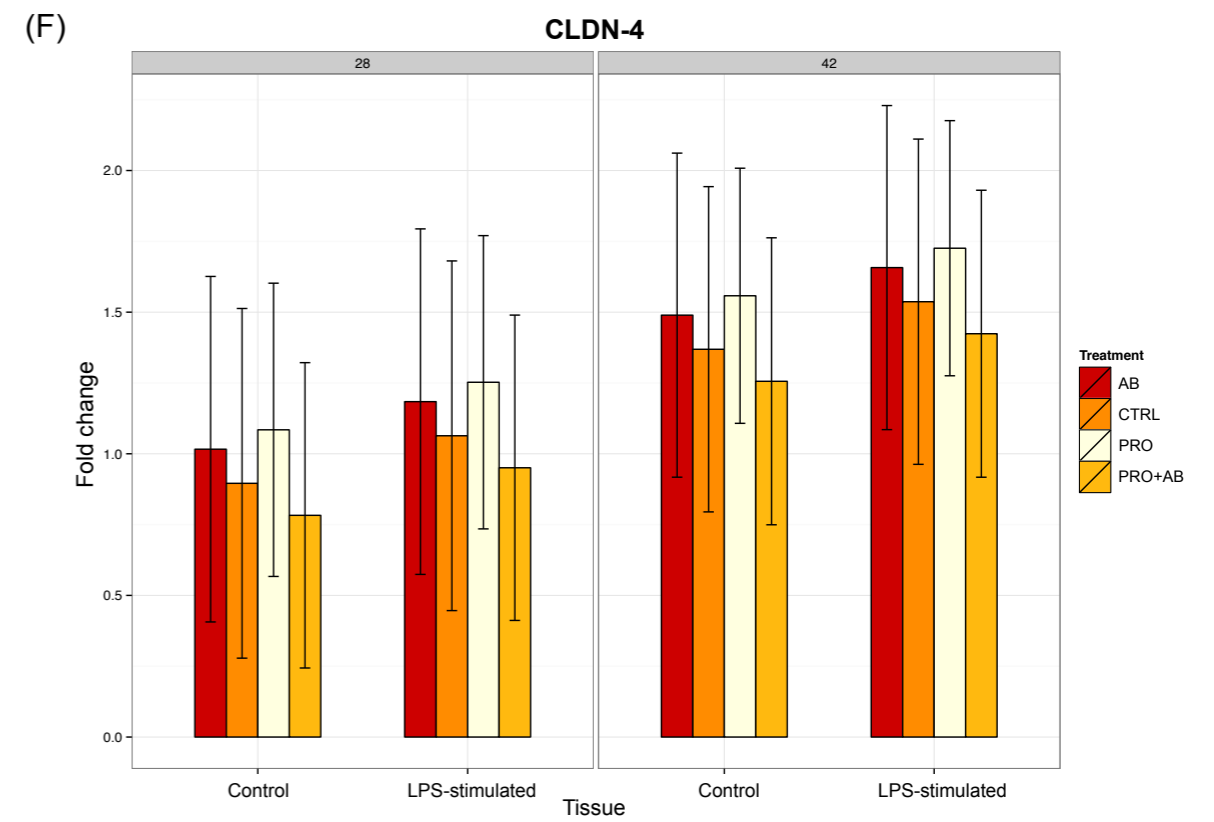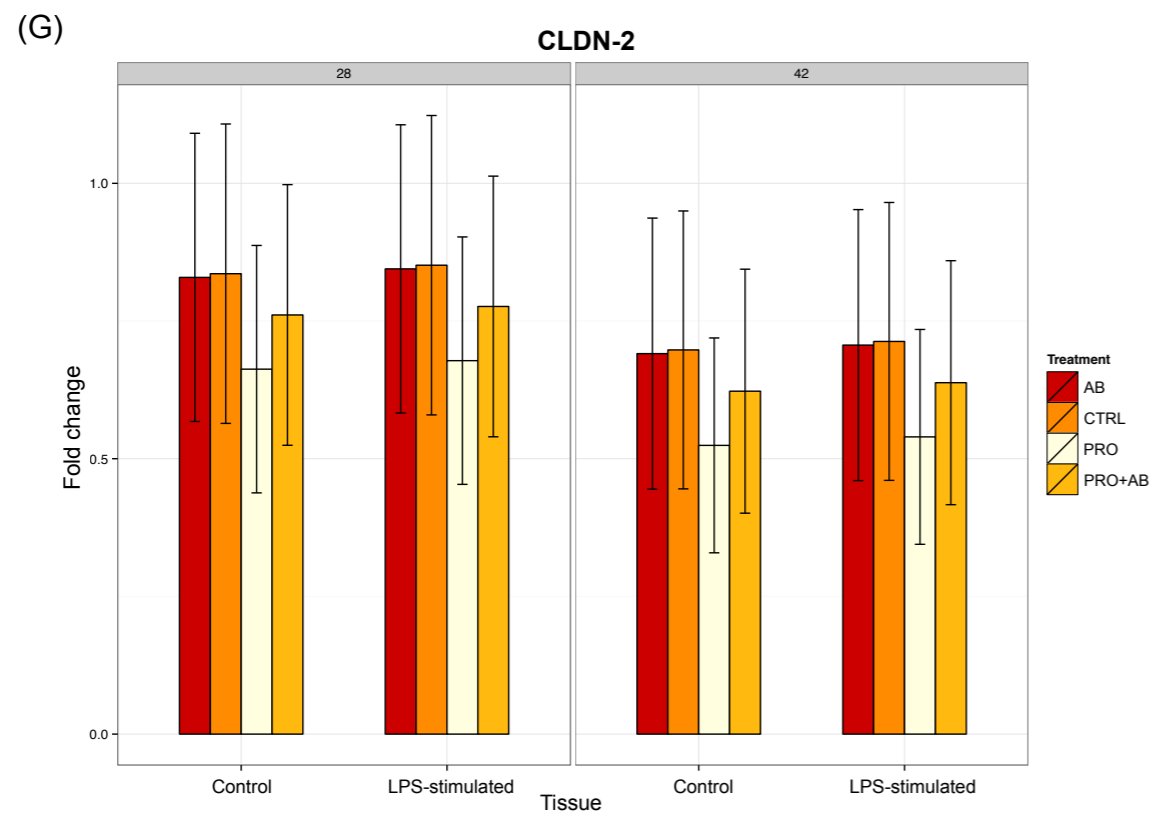

Supplement: S6 Fig — Gene expression levels of (A) TNF-α, (B) IL-10, (C) Cyclo-oxygenase-2, (D) ZO-1, (E) OCLN, (F) CLDN-4, and (G) CLDN-2 in ileal tissue collected day 28 (n = 22) and 42 (n = 34) from piglets administered gentamicin (AB); piglets administered Bacillus spores (PRO); piglets administered both gentamicin and Bacillus spores (PRO+AB); and control piglets not receiving gentamicin or Bacillus spores (CONTROL), that have either been left untreated or stimulated with LPS. Values are presented as least-square means and the 95% confidence intervals presented as vertical bars. Bars embraced by horisontal brackets market by * (0.01≤p<0.05), ** (0.001≤p<0.01) or *** (p<0.001) are significantly different. (PDF) [file pone.0207382.s009.pdf]
